# Hormonal milieu influences whole-brain structural dynamics across the menstrual cycle using dense sampling in multiple individuals

**DOI:** 10.1038/s41593-025-02066-2

**Published:** 2025-09-26

**Authors:** Carina Heller, Daniel Güllmar, Lejla Colic, Laura Pritschet, Martin Gell, Nooshin Javaheripour, Feliberto de la Cruz, Philine Rojczyk, Carina J. Koeppel, Bart Larsen, Habib Ganjgahi, Frederik J. Lange, Ann-Christine Buck, Tim L. Jesgarzewsky, Robert Dahnke, Michael Kiehntopf, Emily G. Jacobs, Zora Kikinis, Martin Walter, Ilona Croy, Christian Gaser

**Affiliations:** 1 Department of Psychiatry and Psychotherapy, Jena University Hospital, Jena, Germany; 2 Masonic Institute for the Developing Brain, University of Minnesota, Minneapolis, MN USA; 3 Department of Pediatrics, University of Minnesota, Minneapolis, MN USA; 4 Department of Psychological and Brain Sciences, University of California, Santa Barbara, Santa Barbara, CA USA; 5 German Center for Mental Health (DZPG), Jena–Magdeburg–Halle, Germany; 6 Center for Intervention and Research on Adaptive and Maladaptive Brain Circuits Underlying Mental Health (C-I-R-C), Jena–Magdeburg–Halle, Germany; 7 Medical Physics Group, Institute of Diagnostic and Interventional Radiology, Jena University Hospital, Jena, Germany; 8 Department of Psychiatry, University of Pennsylvania, Philadelphia, PA USA; 9 Department of Psychiatry and Behavioral Sciences, University of Minnesota, Minneapolis, MN USA; 10 Institute of Neuroscience and Medicine (INM-7: Brain & Behaviour), Research Centre Jülich, Jülich, Germany; 11 Lab for Autonomic Neuroscience, Imaging and Cognition (LANIC), Department of Psychosomatic Medicine and Psychotherapy, Jena University Hospital, Jena, Germany; 12 cBRAIN, Department of Child and Adolescent Psychiatry, Psychosomatics, and Psychotherapy, Ludwig-Maximilians-University, Munich, Germany; 13 Psychiatry Neuroimaging Laboratory, Department of Psychiatry, Brigham and Women’s Hospital, Harvard Medical School, Boston, MA USA; 14 Department of Psychiatry and Neurosciences, Charité – Universitätsmedizin Berlin, corporate member of Freie Universität Berlin and Humboldt-Universität zu Berlin, Berlin, Germany; 15 Institute of Child Development, Univeristy of Minnesota, Minneapolis, MN USA; 16 Big Data Institute, Li Ka Shing Centre for Health Information and Discovery, Nuffield Department of Population Health, University of Oxford, Oxford, UK; 17 Department of Statistics, University of Oxford, Oxford, UK; 18 Oxford University Centre for Integrative Neuroimaging, FMRIB, Nuffield Department of Clinical Neurosciences, University of Oxford, Oxford, UK; 19 Department of Clinical Psychology, Friedrich Schiller University Jena, Jena, Germany; 20 Department of Neurology, Jena University Hospital, Jena, Germany; 21 Institute of Clinical Chemistry and Laboratory Diagnostics, Jena University Hospital, Jena, Germany; 22 Neuroscience Research Institute, University of California, Santa Barbara, Santa Barbara, CA USA; 23 Department of Psychotherapy and Psychosomatic Medicine, University Hospital Carl Gustav Carus, Technische Universität Dresden, Dresden, Germany

**Keywords:** Neuroscience, Brain, Neuroendocrine diseases

## Abstract

Gonadal hormone receptors are widely distributed across the brain, yet their influence on brain structure remains understudied. Here, using precision imaging, we examined four females, including one with endometriosis and one using oral contraceptives (OC), across a monthly period. Whole-brain analyses revealed spatiotemporal patterns of brain volume changes, with substantial variations across the monthly period. In typical cycles, spatiotemporal patterns were associated with serum progesterone levels, while in cycles with endometriosis and during OC intake, patterns were associated with serum estradiol levels. The volume changes were widely distributed rather than region-specific, suggesting a widespread but coordinated influence of hormonal fluctuations. These findings underscore the importance of considering diverse hormonal milieus beyond typical menstrual cycles in understanding structural brain dynamics and suggest that hormonal rhythms may drive widespread structural brain changes.

## Abstract (web summary)

Heller et al. showed dense longitudinal imaging in four females, including one with endometriosis and one using oral contraceptives, and the finding that different hormonal milieus influence widespread brain volume changes linked to progesterone or estradiol.

## Main

Physiological fluctuations in levels of gonadal hormones, such as endogenous estradiol and progesterone, orchestrate the rhythm of the female menstrual cycle throughout the reproductive years^1,2^. The typical menstrual cycle spans 25–32 days, beginning with the follicular phase characterized by menses, followed by a rise in estradiol levels alongside low progesterone concentrations; around cycle day 14, ovulation marks the transition into the luteal phase, marked by rising progesterone levels and a second peak in estradiol, and then followed by a decline in both hormones toward the end of the cycle^3^. Ex vivo animal data have shown a widespread distribution of both progesterone and estradiol receptors throughout the brain, with varying expression levels depending on the specific brain region. While brain structures typically associated with the limbic system (for example, thalamus, hippocampus, amygdala and hypothalamus) are richer in estrogen and progesterone receptors, these receptors are also expressed, albeit to a lesser extent, in the cerebral and cerebellar cortex^4,5^. Estradiol and progesterone have pivotal roles in synaptogenesis, myelination processes and the modulation of spine density^6–11^. As such, these hormones have potential to modulate brain structure, function, chemistry^12–14^ and, by extension, to influence behavior^11^. This is further demonstrated by hormonal influences on cognition, memory^15–18^, stress responsiveness^19–21^ and mood regulation^22–25^. While animal studies have provided valuable insights into the role of gonadal hormones on the brain, they often focus on a limited number of regions (for example, hippocampus). However, given that estradiol and progesterone receptors are expressed across the entire brain, a whole-brain approach is essential to better understand the broader impact of these hormones. Because these hormones can modulate brain structure, examining the entire brain would offer a deeper understanding of their effects on neural dynamics.

Studies investigating the effect of endogenous hormones on brain neuroplasticity in vivo in human neuroscience often involve collecting data from multiple individuals at a single time point to establish mean comparisons and average hormone–brain associations. This cross-sectional method, such as comparing females in the follicular versus the luteal phase, has identified differences in global gray matter volume^26^ as well as region-specific differences (for example, hippocampus^27,28^, parahippocampal gyrus^28,29^, middle frontal gyrus^27,30^ and cerebellum^28^). However, this method overlooks the rhythmic nature of hormone production within the body. Furthermore, averaging across participants may obscure individual differences, warranting a personalized (within-participant) analysis.

Recent years have witnessed a paradigm shift in neuroimaging studies, with an alternative approach that involves the longitudinal tracking of individual participants over extended periods of weeks and months, increasing the sensitivity to detect associations among fluctuations in gonadal hormones and brain structure^31,32^. An emerging trend has centered on the comprehensive monitoring of the female menstrual cycle over time periods ranging from days to weeks and months, aiming to enhance our understanding of hormone-induced effects within the human brain^33–40^. This approach has enriched our insights into the multifaceted impact of hormones on human brain function and structure by detecting subtle changes that could be overlooked in less frequent sampling. Densely sampled neuroimaging studies tracking a single individual across a complete menstrual cycle have primarily focused on investigating functional networks and connectivity^33,35–38^. Only two densely sampled neuroimaging studies have examined structural changes, exclusively within regions of interest (hippocampus and medial temporal lobe^39,40^). In the most recent investigation, 27 female participants underwent six scans throughout their menstrual cycle. Here the authors reported associations among plasma estradiol levels, progesterone levels and subfield volumes within the hippocampus^41^. While region-specific analyses reveal how particular brain areas differ across the menstrual cycle, they do not provide insights into the dynamic changes that occur throughout the brain. Adopting a whole-brain approach would provide a broader perspective on the *range* of brain structures that change across the full menstrual cycle in response to day-to-day hormonal fluctuations.

To expand our understanding of the impact of estradiol and progesterone on the brain’s structure, it is essential to expand the scope of our research beyond individuals with typical menstrual cycle patterns. Including participants with endocrine disorders such as endometriosis, a condition characterized by a unique hormonal profile^42–44^, will provide a more nuanced understanding of the complex interplay between gonadal hormones and their influence on brain structure. Endometriosis, a chronic and inflammatory gynecological disorder, affects approximately 10–15% of females in their reproductive years^45^. It is defined by the presence and growth of ectopic endometrial stroma and glands outside the uterine cavity, typically within the peritoneal cavity. This pathological phenomenon can have various clinical manifestations, including chronic pelvic pain, dysmenorrhea, dyspareunia and infertility^46,47^. The condition is accompanied by hormonal dysregulations—the development, growth and maintenance of endometriotic lesions are driven and sustained by endogenous estrogen, and endometriosis is associated with an increased estradiol synthesis and decreased inactivation, resulting in elevated local concentrations of this hormone, referred to as estrogen dependency^42,43,48–50^. In parallel, the endometriotic lesions can become resistant to the inhibitory actions of endogenous progesterone, known as progesterone resistance^44^; consequently, even in the presence of progesterone, these tissues may continue to grow, bleed and cause inflammation rather than responding with the typical growth suppression seen in healthy endometrial tissue^51–53^.

The current study used four densely sampled females who underwent extensive and standardized brain imaging and venipuncture throughout their entire menstrual cycle. Using a whole-brain approach, we aimed to delineate individualized trajectories of structural brain patterns and to investigate the impact of endogenous day-to-day hormone fluctuations on these trajectories. Similar to the principles of whole-brain functional connectivity analyses, which probe the interactions and communication between different regions, we aim to understand how the brain changes as a whole across the menstrual cycle. Through this approach, we seek to elucidate the influence of hormonal fluctuations on the entire brain, offering nuanced insights into the dynamic processes of hormone-induced neuroplasticity.

**Extended Data Fig. 1:**
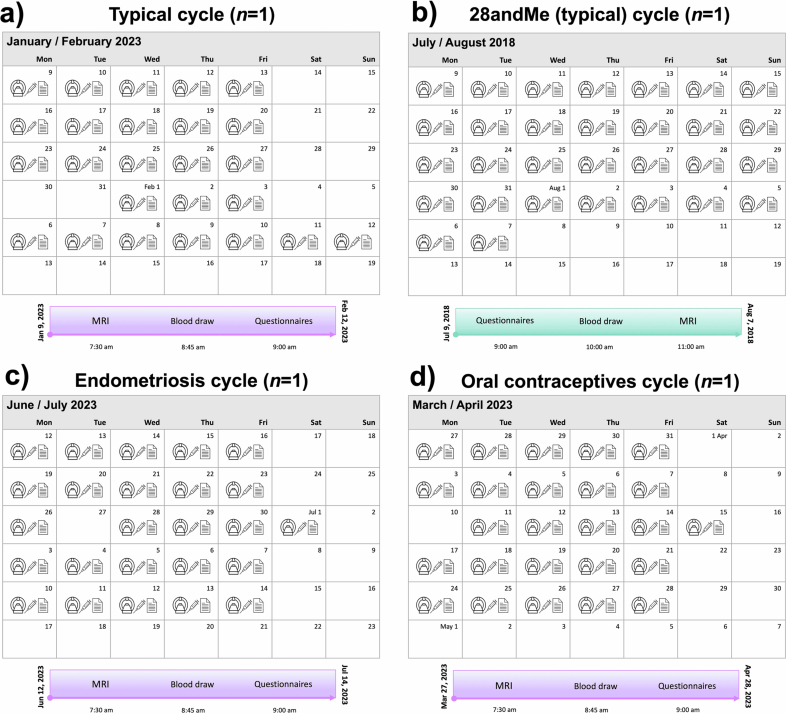
Timeline of the data collection for the female participants (*n* = 4). (**a**) The timeline of data collection for the typical cycle (*n* = 1). (**b**) The timeline of data collection for the 28andMe (typical) cycle (*n* = 1). (**c**) The timeline of data collection for the endometriosis cycle (*n* = 1). (**d**) The timeline of data collection for the oral contraceptives cycle (*n* = 1). For (**a**–**d**) MRI (MRI symbol), blood draw for hormonal assessments (syringe symbol), and mood questionnaires (paper symbol) were acquired simultaneously on each test day. Purple timeline bars represent the endometriosis cycle, the oral contraceptives cycle, and the typical cycle acquired in Jena, Germany. The turquoise timeline bar represents the 28andMe (typical) cycle acquired in Santa Barbara, California, USA.

To investigate neurostructural dynamics across hormonal states, we conducted a dense-sampling study involving multiple participants. First, we densely sampled a healthy female with a typical menstrual cycle, referred to as ‘typical cycle’ (Extended Data Fig. 1a). We then leveraged the densely sampled open-access 28andMe dataset of another female^33–38,40^. This dataset will be referred to as ‘28andMe (typical) cycle’ (Extended Data Fig. 1b). To extend the relevance of our findings and to probe the neural effects of hormonal dysregulation, we repeated these procedures in a female participant diagnosed with endometriosis. This dataset will be referred to as ‘endometriosis cycle’ (Extended Data Fig. 1c). Additionally, we included one female using oral contraceptives (OC), characterized by substantially suppressed endogenous serum progesterone levels, and estradiol levels comparable to a natural cycle. This dataset will be referred to as ‘OC cycle’ (Extended Data Fig. 1d). We first compared endogenous gonadal hormones—serum estradiol levels, serum progesterone levels and their ratio—among the four individuals to evaluate the presence of hormonal dysregulation in the female with endometriosis. Then, using singular value decomposition (SVD) analyses, we generated whole-brain volumetric (VSTPs) and cortical thickness spatiotemporal patterns (CSTPs) across the monthly period. After this, we investigated the potential association between these patterns and gonadal hormones within each individual. Subsequently, voxel-wise and vertex-wise analyses were used to directly link the hormonal fluctuations to structural brain measures. To further contextualize our results, we repeated the study procedure and acquired an additional dense-sampling dataset from one male over a comparable monthly period, during which no specific gonadal hormone patterns were expected.

In this study, we use the term ‘females’ instead of ‘women’ to emphasize the biological aspect, focusing on biological sex rather than gender. It is worth noting that language regarding these terms is constantly evolving. We emphasize that sex hormones represent crucial biological factors in the human experience, transcending any perceived binaries.

## Results

### Endocrine assessments and menstrual cycle patterns

**Fig. 1:**
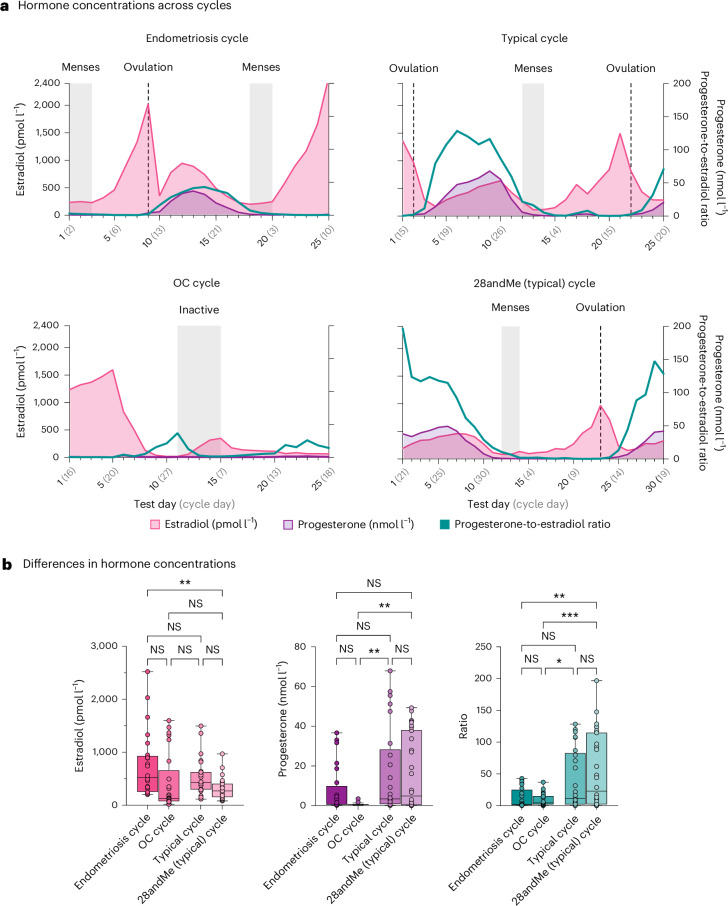
Hormone concentrations of estradiol, progesterone and the progesterone-to-estradiol ratio for female participants (*n* = 4). **a**, Hormones concentrations across the test sessions for female participants (*n* = 4). Solid lines and colored shaded areas represent hormonal levels; gray shading indicates menses in typical cycles and the endometriosis cycle, and inactive pill phase in the oral contraceptives (OC) cycle; dashed lines indicate ovulation. Hormone levels indicate a typical hormonal balance in the typical and 28andMe (typical) cycles, while hormone levels in the endometriosis and OC cycles suggest estradiol dominance. **b**, To test whether hormonal profiles differed among the four individuals, a one-way MANOVA was conducted, followed by a post hoc ANOVAs, and two-sided post hoc *t*-tests. The box-and-whisker plots show the median (centerline), upper and lower quartiles (box), minimum and maximum values (whiskers); individual points are shown. Asterisks indicate significance level (***P < 0.001, **P < 0.01, *P < 0.05) based on two-sided post hoc *t*-tests with Bonferroni correction for multiple comparisons. For exact *P* values, see Main. Graphs were created with GraphPad Prism (version 10). NS, nonsignificant; MANOVA, multivariate analysis of variance; ANOVAs, analyses of variance. Source data

Gonadal hormones were assessed throughout the full menstrual cycle (Fig. 1a). Analyses of hormone serum concentrations in the typical cycle and the 28andMe (typical) cycle confirmed the expected rhythmic changes of a natural menstrual cycle. In the typical cycle, the 25 test sessions covered 15 days of the follicular phase and 10 days of the luteal phase. In the 28andMe (typical) cycle, the 30 test sessions covered 14 days of the follicular phase and 16 days of the luteal phase. The ratios between progesterone and estradiol concentrations suggested a typical hormonal balance during the luteal phase.

In the endometriosis cycle, gonadal hormone concentrations also followed the rhythmic changes of a menstrual cycle. The 25 test sessions covered 17 days of the follicular phase and 8 days of the luteal phase. As predicted, the progesterone-to-estradiol ratio suggested an estradiol dominance during the luteal phase. The menstrual cycles covered in the participant with endometriosis lasted 23 and 24 days respectively during the experiment, representing shorter menstrual cycles (≤24 days)—a typical feature of endometriosis. In the OC cycle, circulating progesterone levels were selectively suppressed. The concentration and dynamic range of estradiol during the oral contraception intake were similar to those observed in a typical menstrual cycle. Progesterone-to-estradiol ratios suggested an estradiol dominance, providing an additional dataset with a hormonal milieu similar to that of the endometriosis cycle.

Progesterone concentrations surpassed 15.9 nmol l^−1^ in the typical, 28andMe (typical) and endometriosis cycle, suggesting an ovulatory cycle^54^.

To test whether hormonal profiles differed between participants, a one-way multivariate analysis of variance was conducted with serum estradiol levels, progesterone levels and the progesterone-to-estradiol ratio as dependent variables, and the four individuals (typical cycle, 28andMe (typical) cycle, endometriosis cycle and OC cycle) as fixed factors. The analysis revealed a significant main effect among the four individuals (Pillai’s trace, *F*_(9,300)_ = 4.52, *P* < 0.001 *η*^2^ = 0.12; Roy’s largest root, *F*_(3,100)_ = 10.14, *P* < 0.001, *η*^2^ = 0.23). Post hoc analyses of variance indicated significant differences among the four individuals in estradiol levels (*F*_(3,100)_ = 4.70, *P* = 0.004, *η*^2^ = 0.12), progesterone (*F*_(3,100)_ = 5.94, *P* < 0.001, *η*^2^ = 0.15) and the progesterone-to-estradiol ratio (*F*_(3,100)_ = 7.83, *P* < 0.001, *η*^2^ = 0.19). Post hoc two-tailed *t*-tests, corrected using the Bonferroni method, further revealed that the endometriosis cycle had significantly higher estradiol levels compared to the 28andMe (typical) cycle (*P* = 0.002), and that progesterone levels were significantly lower in the OC cycle compared to the typical cycle (*P* = 0.005) and the 28andMe (typical) cycle (*P* = 0.003). The endometriosis cycle also showed a significantly lower progesterone-to-estradiol ratio compared to the 28andMe (typical) cycle (*P* = 0.002), and the OC showed lower progesterone-to-estradiol ratios compared to both the typical cycle (*P* = 0.045) and the 28andMe (typical) cycle (*P* < 0.001). Differences in hormonal values are displayed in Fig. 1b.

### Whole-brain structural dynamics

T_1_-weighted (T1w) images were acquired from each participant across the full menstrual cycle—five consecutive weeks for the typical cycle, the endometriosis cycle and the OC cycle, and four consecutive weeks for the 28andMe (typical) cycle. Preprocessing was performed using SPM12 (http://www.fil.ion.ucl.ac.uk/spm) and CAT12 toolbox (https://neuro-jena.github.io/cat)^55^ with the longitudinal pipeline approach. Each processed T1w image represents a snapshot of brain structure on each test day. Next, SVD analysis was used to extract VSTPs and CSTPs. SVD decomposed the images into spatiotemporal components, reflecting patterns of brain structure over time. To capture shared spatial patterns across individuals, data from all cycles (typical, 28andMe (typical), endometriosis and OC cycle) were concatenated.

**Fig. 2:**
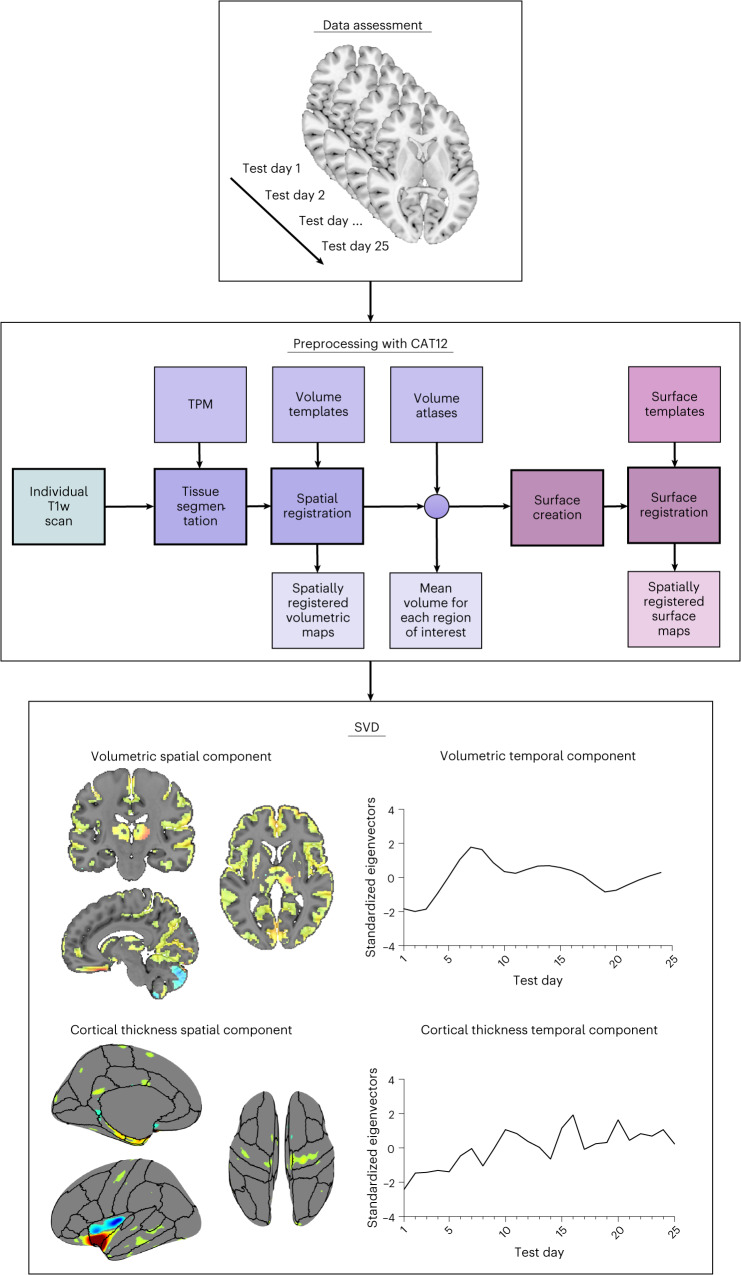
Schematic illustration of data assessment, processing workflow and data reduction. T1w images were assessed over a 4–5-week period for each participant. Images were then preprocessed using the longitudinal pipeline approach in CAT12. Next, SVD was applied to decompose the preprocessed images into spatial and temporal components. Spatial components represent changes in brain volumes and cortical thickness across different regions, while temporal components reflect how these spatial components evolve over time. Warm and cool colors in the spatial component represent positive (warm colors) and negative (cool colors) associations between spatial components and temporal patterns. This suggests that regions marked in warm colors increase as the associated temporal pattern increases, while those in cool colors decrease. Note that the spatial and temporal components shown are examples and do not represent actual results. Graphs were created with GraphPad Prism (version 10). SVD, singular value decomposition; TPM, tissue probability maps.

In SVD, eigenvalues represent the variance explained by each principal component, while eigenvectors represent the temporal patterns. Warm colors in the spatial components denote positive associations with the eigenvectors of the temporal component, indicating that these regions increase as the corresponding temporal pattern increases. Cool colors signify negative associations, meaning these regions decrease as the temporal pattern increases. These patterns are referred to as ‘spatiotemporal patterns’. It is important to note that the values derived from the SVD (eigenvalues and eigenvectors) are arbitrary in magnitude, meaning they lack an inherent unit of measurement but are used to identify patterns of association. A schematic illustration of the workflow is shown in Fig. 2.

#### Volumetric dynamics

**Fig. 3:**
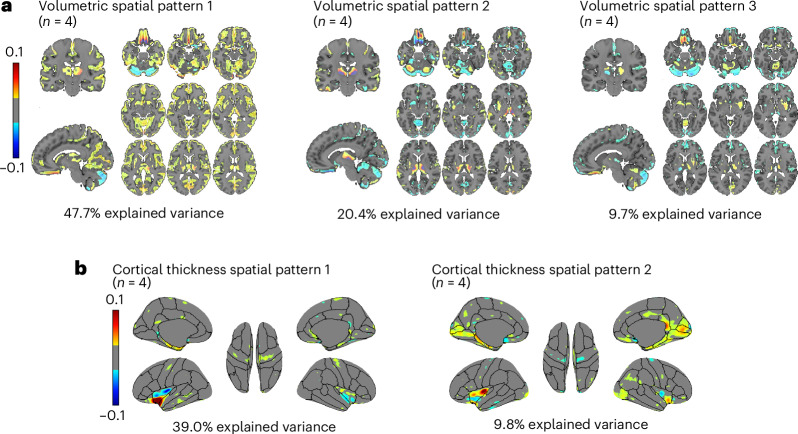
Volumetric and cortical thickness spatial patterns that explained at least 10% of the variance across the female participants (*n* = 4). **a**, The spatial patterns illustrate the volumetric patterns of involved brain regions that change over time across the female participants (*n* = 4; the endometriosis, oral contraceptives (OC), typical and 28andMe (typical) cycle). **b**, The spatial patterns illustrate the cortical thickness patterns of involved brain regions that change over time across the female participants (*n* = 4; the endometriosis, OC, typical and 28andMe (typical) cycle). For **a** and **b**, volumetric and cortical thickness spatial patterns were derived using SVD. Spatial weights were thresholded, retaining only values within the ranges of −0.1 to −0.01 and 0.01 to 0.1, while excluding values between −0.01 and 0.01 that indicate minimal contribution to the respective spatial pattern (color bar). Source data

In the volumetric analysis (Fig. 3a), VSTP1 explained 47.7% of the variance, with the most substantial clusters spanning the gray matter of the cerebellum, precuneus, middle frontal gyrus, lingual gyrus, angular gyrus and temporal gyrus. VSTP2 explained 20.4% of the variance, with the most substantial clusters overlapping with the gray matter of the cerebellum, thalamus, temporal gyrus, precentral gyrus and gyrus rectus. VSTP3 explained 9.7% of the variance, with the most substantial clusters located in the gray matter of the cerebellum, superior and middle frontal gyrus, supplementary motor cortex, precuneus, precentral gyrus and thalamus (Supplementary Table 1). All other VSTPs explained less than 10% of the variance and were excluded from further analyses.

**Fig. 4:**
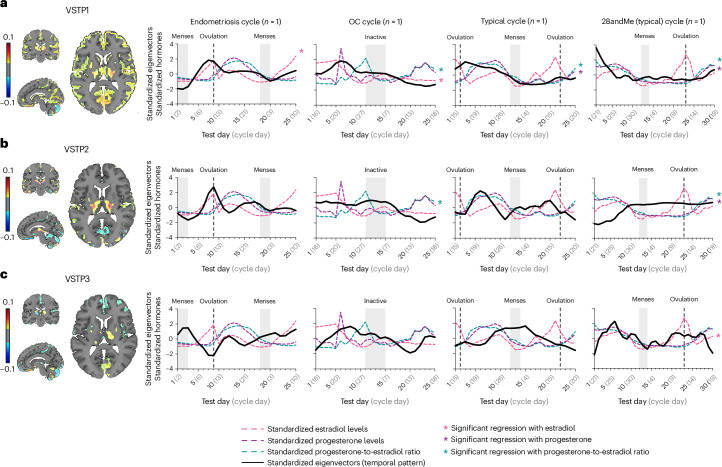
VSTPs across the different female cycles (*n* = 4). This figure depicts VSTPs across the endometriosis cycle, the oral contraceptives (OC) cycle, the typical cycle and the 28andMe (typical) cycle. **a**, VSTP1 shows spatial distribution of brain regions involved in component 1 (left) and the associated temporal dynamics (right). Warm colors in the spatial map indicate regions with positive associations to the temporal pattern (indicating regional volume increases as the temporal pattern increases). Cool colors in the spatial map indicate negative associations to the temporal pattern (reflecting regional volume decreases as the temporal pattern increases). **b**, VSTP2 shows spatial distribution of brain regions involved in component 2 (left) and the associated temporal dynamics (right). Warm colors in the spatial map indicate regions with positive associations to the temporal pattern (indicating regional volume increases as the temporal pattern increases). Cool colors in the spatial map indicate negative associations to the temporal pattern (reflecting regional volume decreases as the temporal pattern increases). **c**, VSTP3 shows spatial distribution of brain regions involved in component 3 (left) and the associated temporal dynamics (right). Warm colors in the spatial map indicate regions with positive associations to the temporal pattern (indicating regional volume increases as the temporal pattern increases). Cool colors in the spatial map indicate negative associations to the temporal pattern (reflecting regional volume decreases as the temporal pattern increases). For **a**–**c**, volumetric and cortical thickness spatial patterns were derived using SVD. Spatial weights were thresholded, retaining only values within the ranges of −0.1 to −0.01 and 0.01 to 0.1, while excluding values between −0.01 and 0.01 that indicate minimal contribution to the respective spatial pattern (color bar). Solid black lines represent standardized eigenvectors (temporal pattern); dashed colored lines represent square-rooted and standardized hormonal values; gray shading indicates menses in typical cycles and the endometriosis cycle, and inactive pill phase in the OC cycle; dashed lines indicate ovulation. Asterisks indicate significant time-series regressions between hormone levels and the spatiotemporal patterns after FDR correction for multiple comparisons was performed. For exact *P* values, see main text. Plots were created with GraphPad Prism (version 10). VSTPs, volumetric spatiotemporal patterns. Source data

Generalized additive models (GAMs) were used to analyze changes in the extracted spatiotemporal patterns across the monthly period. This choice was motivated by its ability to capture potential nonlinear trends, including curvature and variations in change rates, that are often present in the longitudinal data. VSTP1, VSTP2 and VSTP3 were found to significantly fluctuate across all four participants (Fig. 4 and Supplementary Table 2).

#### Hormonal associations with volumetric dynamics

To assess whether the short-term VSTPs were driven by fluctuations in gonadal hormones, time-series regression analyses were used. Serum estradiol levels, progesterone levels and the progesterone-to-estradiol ratio were separately specified as independent variables for each individual and spatiotemporal pattern. Because not all variables were normally distributed, relationships were further modeled using nonparametric functional Spearman rank correlation. Results were highly consistent across both approaches.

In the typical cycle, both progesterone levels (*β* = 0.021, *P*_FDR_ = 0.010) and the progesterone-to-estradiol ratio (*β* = 0.015, *P*_FDR_ = 0.007) were significantly associated with VSTP1, with corresponding significant Spearman correlations (progesterone, *ρ* = 0.642, *P*_FDR_ = 0.005; progesterone-to-estradiol ratio, *ρ* = 0.587, *P*_FDR_ = 0.011). Similarly, for the 28andMe (typical) cycle, progesterone levels (*β* = 0.017, *P*_FDR_ < 0.001) and the progesterone-to-estradiol ratio (*β* = 0.011, *P*_FDR_ < 0.001) showed positive associations with VSTP1, again with corresponding significant Spearman correlations (progesterone, *ρ* = 0.586, *P*_FDR_ = 0.002; progesterone-to-estradiol ratio, *ρ* = 0.693, *P*_FDR_ < 0.001). Additionally, progesterone levels (*β* = −0.044, *P*_FDR_ < 0.001) and the progesterone-to-estradiol ratio (*β* = −0.025, *P*_FDR_ < 0.001) were significantly negatively associated with VSTP2 (*P*_FDR_ < 0.001), supported by corresponding negative Spearman correlations (progesterone, *ρ* = −0.631, *P*_FDR_ < 0.001; progesterone-to-estradiol ratio, *ρ* = −0.592, *P*_FDR_ = 0.002). Estradiol levels were significantly associated with VSTP3 only in the 28andMe (typical) cycle (*β* = 0.003, *P*_FDR_ < 0.044), supported by a corresponding Spearman correlation (*ρ* = 0.571, *P*_FDR_ = 0.002).

In the endometriosis cycle, estradiol levels were significantly associated with VSTP1 (*β* = 0.006, *P*_FDR_ = 0.010), with a corresponding significant Spearman correlation (*ρ* = 0.571, *P*_FDR_ = 0.037). No significant relationships were observed for VSTP2 or VSTP3. Similarly, in the OC cycle, estradiol levels showed a significant association with VSTP1 (*β* = 0.006, *P*_FDR_ = 0.023). Additionally, the progesterone-to-estradiol ratio was significantly negatively associated with VSTP1 (*β* = −0.037, *P*_FDR_ = 0.046) and VSTP2 (*β* = −0.021, *P*_FDR_ = 0.046). However, Spearman correlations in the OC cycle did not remain significant after false discovery rate (FDR) correction. No significant associations were observed for VSTP3. Progesterone levels did not exhibit significant associations in either the endometriosis or the OC cycle (Fig. 4). All results are displayed in Supplementary Table 3.

#### Cortical thickness dynamics

In the cortical thickness analysis (Fig. 3b), CSTP1 explained 39.0% of the variance, with the largest clusters spanning the insula, precentral gyrus and superior temporal gyrus. CSTP2 explained 9.8% of the variance, with the largest clusters spanning the insula, lingual gyrus, lateral occipital lobe, pericalcarine gyrus, parahippocampal gyrus and fusiform gyrus (Supplementary Table 4). All other CSTPs explained less than 10% of the variance and were excluded from further analyses. GAMs revealed that CSTP2 exhibited substantial fluctuations only in the 28andMe (typical) cycle (Supplementary Table 5), and no significant fluctuations were observed in CSTP1 in any participant.

#### Hormonal associations with cortical thickness dynamics

**Fig. 5:**
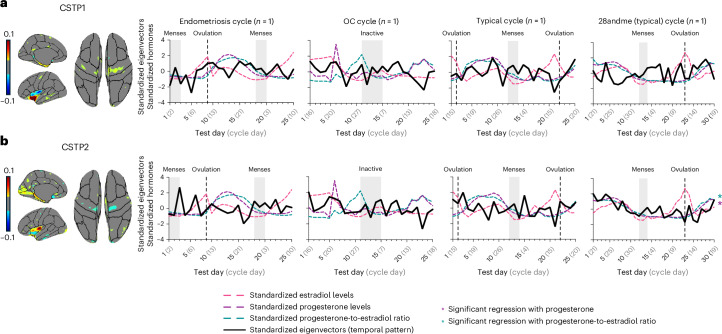
CSTPs across the different female cycles (*n* = 4). This figure depicts CSTPs across the endometriosis cycle, the OC cycle, the typical cycle and the 28andMe (typical) cycle. **a**, CSTP1 shows spatial distribution of brain regions involved in component 1 (left) and the associated temporal dynamics (right). Warm colors in the spatial map indicate regions with positive associations to the temporal pattern (indicating regional cortical thickness increases as the temporal pattern increases). Cool colors in the spatial map indicate negative associations to the temporal pattern (reflecting regional cortical thickness decreases as the temporal pattern increases). **b**, CSTP2 shows spatial distribution of brain regions involved in component 2 (left) and the associated temporal dynamics (right). Warm colors in the spatial map indicate regions with positive associations to the temporal pattern (indicating regional cortical thickness increases as the temporal pattern increases). Cool colors in the spatial map indicate negative associations to the temporal pattern (reflecting regional cortical thickness decreases as the temporal pattern increases). For **a** and **b**, volumetric and cortical thickness spatial patterns were derived using SVD. Spatial weights were thresholded, retaining only values within the ranges of −0.1 to −0.01 and 0.01 to 0.1, while excluding values between −0.01 and 0.01 that indicate minimal contribution to the respective spatial pattern (color bar). Solid black lines represent standardized eigenvectors (temporal pattern); dashed colored lines represent square-rooted and standardized hormonal values; gray shading indicates menses in typical cycles and the endometriosis cycle, and inactive pill phase in the OC cycle; dashed lines indicate ovulation. Asterisks indicate significant time-series regressions between hormone levels and the spatiotemporal patterns after FDR correction for multiple comparisons was performed. For exact *P* values, see main text. Plots were created with GraphPad Prism (version 10). CSTPs, cortical thickness spatiotemporal patterns. Source data

Progesterone levels and the progesterone-to-estradiol ratio were significantly associated with CSPT2 in the 28andMe (typical) cycle only (progesterone, *β* = 0.042, *P*_FDR_ < 0.001; progesterone-to-estradiol ratio, *β* = 0.023, *P*_FDR_ < 0.001), supported by corresponding Spearman correlations (progesterone, *ρ* = 0.593, *P*_FDR_ = 0.002; progesterone-to-estradiol ratio, *ρ* = 0.612, *P*_FDR_ = 0.002). No significant associations were observed between other predictors and CSTP1 or CSTP2 in any of the remaining cycles. All results are displayed in Fig. 5 and Supplementary Table 6.

### Complementary voxel-wise and vertex-wise analyses

To directly link hormonal fluctuations to structural brain measures, complementary voxel-wise and vertex-wise analyses were conducted as a sensitivity check. To confirm the hormone–SVD associations, we repeated the analyses at the voxel level (for volume) and the vertex level (for thickness) to assess whether similar spatial patterns of associations emerged.

**Fig. 6:**
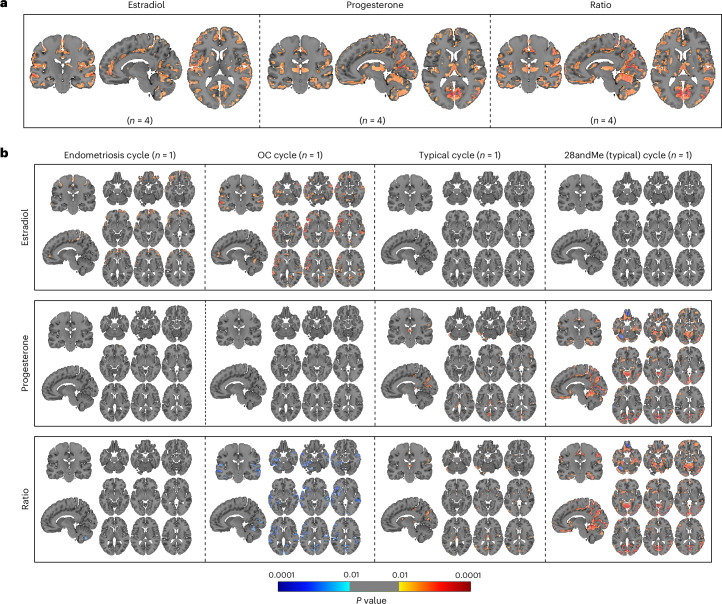
Significant voxels associated with hormone concentrations in the female participants (*n* = 4). **a**, The significant voxel-wise associations across all four cycles (*n* = 4; endometriosis cycle, oral contraceptives (OC) cycle, typical cycle and 28andMe (typical) cycle). **b**, The presentation of the significant voxels for each cycle separately (endometriosis cycle, *n* = 1; OC cycle, *n* = 1; typical cycle, *n* = 1; and 28andMe (typical) cycle, *n* = 1). For **a** and **b**, GLMs were used for vertex-wise analysis with the TFCE method that controls for multiple comparisons by applying an FWE correction. Hormone concentrations were square-rooted. Positive associations are displayed in red, negative associations are displayed in blue, with *P* values ranging from 0.01 to 0.0001 (color bar). GLMs, general linear models; TFCE, threshold-free cluster enhancement; FWE, family-wise error; Ratio, progesterone-to-estradiol ratio. Source data

Voxel-wise analyses revealed widespread positive associations between brain volume and hormonal concentrations of estradiol, progesterone and the progesterone-to-estradiol ratio across all individuals (Fig. 6a). These associations overlapped to some extent with the spatial patterns observed in the SVD analyses. Contrasted analyses indicated that the endometriosis and OC cycles predominantly drove the associations with estradiol levels, while associations with progesterone levels were primarily influenced by the typical and 28andMe (typical) cycles (Fig. 6b). Estradiol levels were mainly positively associated with the cingulate gyrus, frontal gyrus, orbital gyrus, precentral gyrus, superior temporal gyrus and supramarginal gyrus. Progesterone levels and the progesterone-to-estradiol ratio were positively associated with the cerebellum, cuneus, inferior temporal, postcentral and superior parietal gyrus. Regions that were positively associated with both estradiol levels and progesterone levels, as well as the progesterone-to-estradiol ratio, were the precuneus and angular gyrus (Supplementary Table 7). Negative associations were primarily observed in the OC cycle for the progesterone-to-estradiol ratio (Supplementary Table 8).

**Fig. 7:**
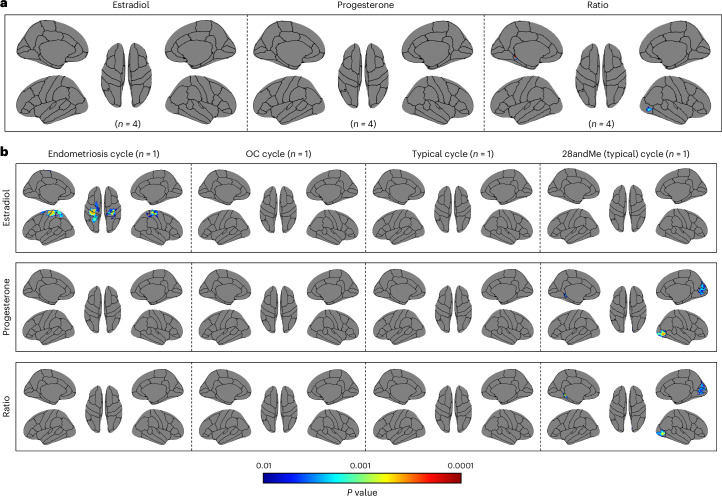
Significant vertices associated with hormone concentrations in the female participants (*n* = 4). **a**, The significant vertex-wise associations across all four cycles (*n* = 4; endometriosis cycle, oral contraceptives (OC) cycle, typical cycle and 28andMe (typical) cycle). **b**, The presentation of the significant vertices for each cycle separately (endometriosis cycle, *n* = 1; OC cycle, *n* = 1; typical cycle, *n* = 1; and 28andMe (typical) cycle, *n* = 1). For **a** and **b**, GLMs were used for vertex-wise analysis with the TFCE method that controls for multiple comparisons by applying an FWE correction. Hormone concentrations were square-rooted. Only positive associations were observed, with *P* values ranging from 0.01 to 0.0001 (color bar). Ratio, progesterone-to-estradiol ratio. Source data

Vertex-wise analyses revealed only a few associations between cortical thickness and hormone concentrations. Significant positive associations were observed between the progesterone-to-estradiol ratio and cortical thickness of the parahippocampal and lateral occipital gyrus across all individuals (Fig. 7a). No significant associations were found with estradiol and progesterone levels. Contrasted analyses revealed significant positive associations between estradiol levels and cortical thickness of the postcentral, superior parietal, precentral and superior frontal gyrus in the endometriosis cycle only. The progesterone-to-estradiol ratio was associated with cortical thickness of the parahippocampal, lingual, lateral occipital, pericalcarine gyrus and cuneus only in the 28andMe (typical) cycle (Fig. 7b). No other significant associations were observed (Supplementary Table 9).

### Comparison to a male participant

**Extended Data Fig. 2:**
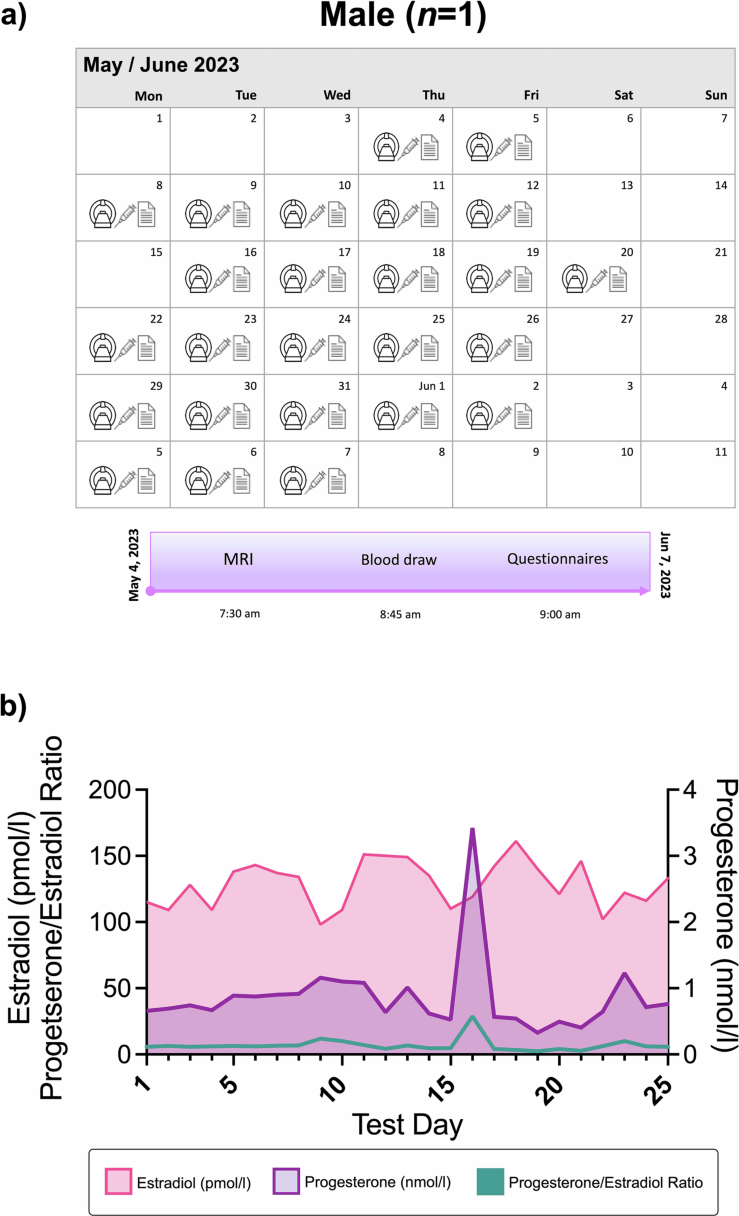
Timeline of data collection and hormonal levels for the male participant (*n* = 1). (**a**) The timeline of data collection for the male participant (*n* = 1). MRI (MRI symbol), blood draw for hormonal assessments (syringe symbol), and mood questionnaires (paper symbol) were acquired simultaneously on each test day. Data was acquired in Jena, Germany. (**b**) The hormonal levels of estradiol, progesterone, and the progesterone-to-estradiol ratio for the male participant across the five-week period. Solid lines and colored shaded areas represent hormonal levels. The male participant presented with anticipated low hormonal concentrations of estradiol, progesterone, and a low progesterone-to-estradiol ratio. Source data

We repeated all analyses in a male participant where no specific gonadal hormone patterns were expected. The male participant was scanned over a comparable 5-week period, resulting in 25 test sessions (Extended Data Fig. 2a). Hormone concentrations were generally low (estradiol—*M* = 128.7 pmol l^−1^, s.d. = 17.3 pmol l^−1^, range = 98.0–161.0 pmol l^−1^; progesterone—*M* = 0.863 nmol l^−1^, s.d. = 0.582 nmol l^−1^, range = 0.329–3.420 nmol l^−1^; ratio—*M* = 6.921, s.d. = 5.049, range = 2.35–28.74; Extended Data Fig. 2b).

**Extended Data Fig. 3:**
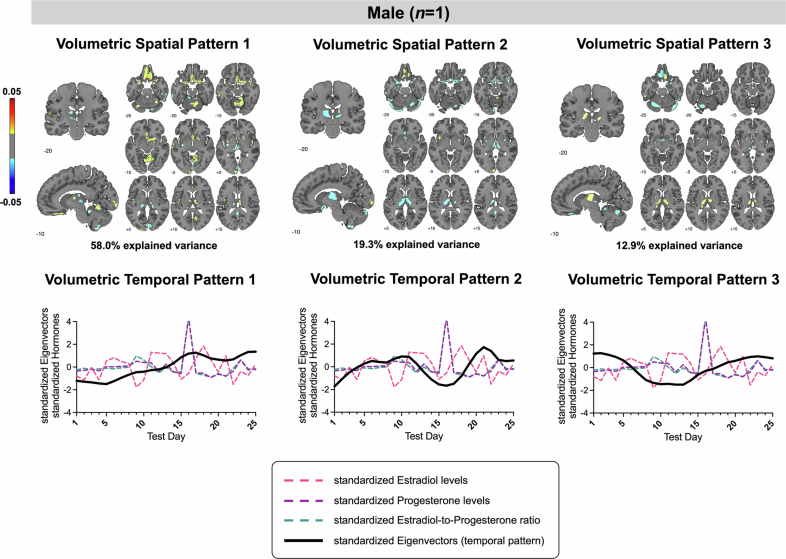
Volumetric spatiotemporal patterns that significantly fluctuated in the male participant (*n* = 1) across the five-week period. This figure depicts volumetric spatiotemporal patterns in the male participant that explained at least 10% of the variance. Spatial distribution of brain regions (top) and the associated temporal dynamics (bottom) of volumetric spatiotemporal pattern 1 (VSTP1), volumetric spatiotemporal pattern 2 (VSTP2) and volumetric spatiotemporal pattern 3 (VSTP3) are shown. Warm colors in the spatial maps indicate regions with positive associations to the temporal pattern (indicating regional volume increases as the temporal pattern increases). Cool colors in the spatial maps indicate negative associations to the temporal pattern (reflecting regional volume decreases as the temporal pattern increases). Spatial weights were thresholded, retaining only values within the ranges of −0.05 to −0.01 and 0.01 to 0.05, while excluding values between −0.01 and 0.01 that indicate minimal contribution to the respective spatial pattern (color bar). Solid black lines represent standardized eigenvectors (temporal pattern); dashed colored lines represent square-rooted and standardized hormonal values. Generalized additive models (GAMs) revealed that the volumetric spatiotemporal patterns fluctuated significantly across time. Time-series regressions revealed that the volumetric temporal patterns were not associated with hormonal levels in the male. For exact p-values, see Supplementary Table 10 and 11. Graphs were created using GraphPad Prism (version 10). Source data

**Extended Data Fig. 4:**
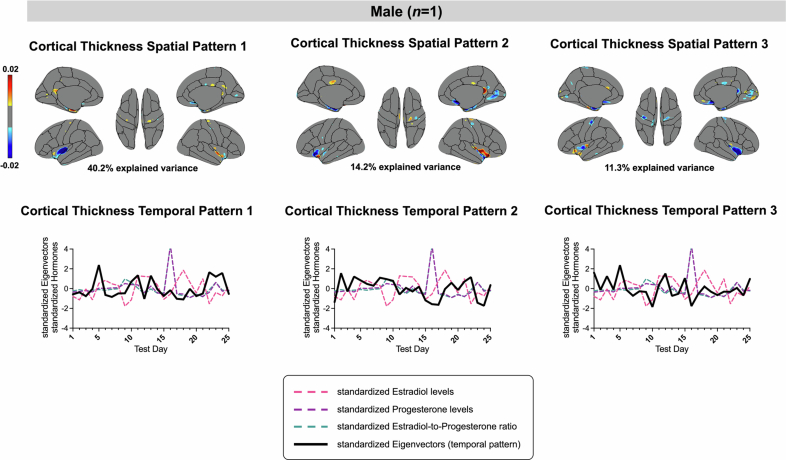
Cortical thickness spatiotemporal patterns in the male participant (*n* = 1) across the five-week period. This figure depicts cortical thickness spatiotemporal patterns in the male participant that explained at least 10% of the variance. Spatial distribution of brain regions (top) and the associated temporal dynamics (bottom) of cortical thickness spatiotemporal pattern 1 (CSTP1), cortical thickness spatiotemporal pattern 2 (CSTP2) and cortical thickness spatiotemporal pattern 3 (CSTP3) are shown. Warm colors in the spatial maps indicate regions with positive associations to the temporal pattern (indicating regional cortical thickness increases as the temporal pattern increases). Cool colors in the spatial maps indicate negative associations to the temporal pattern (reflecting regional cortical thickness decreases as the temporal pattern increases). Spatial weights were thresholded, retaining only those within the ranges of -0.02 to -0.01 and 0.01 to 0.02, while excluding values between -0.01 and 0.01 that indicate minimal contribution to the respective spatial pattern (color bar). Solid black lines represent standardized eigenvectors (temporal pattern); dashed colored lines represent square-rooted and standardized hormonal values. Generalized additive models (GAMs) revealed that the cortical thickness temporal patterns did not significantly fluctuate across the five-week period and time-series regressions revealed that they were not associated with hormonal levels in the male. For exact p-values, see Supplementary Table 10 and 11. Graphs were created using GraphPad Prism (version 10). Source data

VSTP analyses revealed that VSTP1 explained 58.0% of the variance, VSTP2 explained 19.3% of the variance and VSTP3 explained 12.9% of the variance (Extended Data Fig. 3). CSTP analyses revealed that CSTP1 explained 40.2% of the variance, CSTP2 explained 14.2% of the variance and CSTP3 explained 11.3% of the variance (Extended Data Fig. 4). All other volumetric and CSTPs explained less than 10% of the variance and were excluded from further analyses.

**Extended Data Fig. 5:**
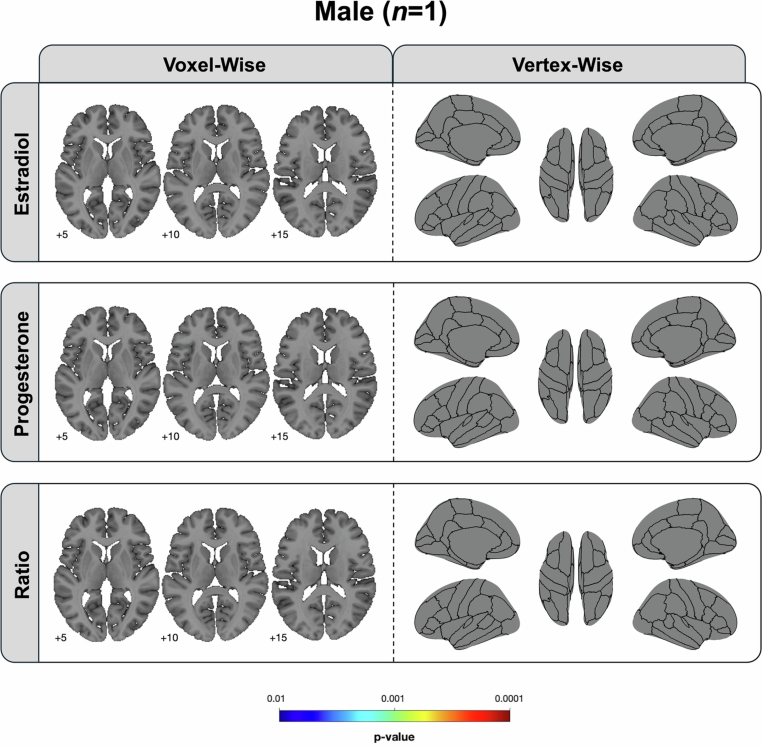
Results from the voxel- and vertex-wise analyses in the male participant (n = 1) across the five-week period. To directly link hormonal fluctuations to structural brain measures, complementary voxel- and vertex-wise analyses were also conducted using general linear models (GLMs) in the male participant as a sensitivity check with the Threshold-Free Cluster Enhancement (TFCE) method which controls for multiple comparisons by applying a family-wise error (FWE) correction. No significant associations were found in the voxel- and vertex-wise analysis between structural brain measures (volume, cortical thickness) and hormone levels in males. Estradiol, square-rooted estradiol levels; progesterone, square-rooted progesterone levels; ratio, square-rooted progesterone-to-estradiol ratio. Source data

While VSTP1–VSTP3 significantly changed across the 5-week period (Supplementary Table 10), no associations were found with either estradiol levels, progesterone levels or the progesterone-to-estradiol ratio (Supplementary Table 11). CSTP1–CSTP3 did not show significant changes across the 5-week period and were not associated with hormone concentrations (Supplementary Tables 10–11). Likewise, the voxel-wise and vertex-wise analyses revealed no significant associations with hormone concentrations (Extended Data Fig. 5).

## Discussion

Despite growing interest in the associations between gonadal hormones and fluctuations in brain structure, whole-brain approaches with broader spatiotemporal resolution are scarce. Such analyses provide insights into how the brain operates synchronously over time. Moreover, investigations into hormone–brain interactions in nontypical cycles—such as those in endometriosis or hormonal contraceptive use—remain understudied. In the present study, we leveraged data from four densely sampled females—two with typical cycles, one with endometriosis and one using OC—and one male, each of whom underwent routine neuroimaging and venipuncture over a monthly period. Using a whole-brain SVD analytical approach, we explored brain structural dynamics across these diverse hormonal conditions. The corresponding datasets are openly available, providing a resource for future investigations into brain plasticity across menstrual cycles and beyond.

While previous precision imaging studies have focused on region-specific analyses^40,41^, here we extend this work by examining whole-brain structural dynamics across the menstrual cycle. Results revealed VSTPs that exhibited substantial variations in all four female individuals across the monthly period. These fluctuations were widespread and distributed across the entire brain. Notably, while these patterns were observed in all four female individuals, the nature and dynamics of how these widespread patterns fluctuated over the monthly period were unique to each individual. Interestingly, the temporal dynamics of the volumetric spatial pattern explaining the most variance were most similar in the endometriosis and OC cycle, which are both characterized by a hormonal milieu dominated by estradiol. In contrast, individuals with typical cycles exhibited more similar temporal dynamics of the volumetric spatial pattern, which explained the most variance, reflecting the cyclical interplay between progesterone and estradiol. Notably, the hormonal correlates of this dominant pattern differed by cycle type—estradiol in the endometriosis and OC cycle, and progesterone in the typical cycles. The association of this pattern with gonadal hormones across all cycles supports the notion that while hormones do have a role in shaping cyclical brain dynamics, not all structural variation across the cycle is hormone-driven and acknowledges the multidimensional nature of brain plasticity.

CSTPs, however, did not fluctuate across individuals, with the exception of the 28andMe (typical) cycle. Cortical thickness analyses inherently exclude the cerebellum and subcortical structures, which have been shown to substantially contribute to the whole-brain SVD patterns observed in volumetric analyses. The cerebellum, as well as subcortical structures, are known to contain sex steroid receptors^4,5^, which may make them particularly sensitive to hormonal fluctuations. The exclusion of these structures in cortical thickness analyses may partly explain why, at the whole-brain level, CSTPs did not exhibit fluctuations across the cycle or show associations with sex steroid hormones. Another explanation for the absence of fluctuations and associations in the cortical thickness measures may lie in the underlying biophysical properties that drive both volumetric and cortical thickness signals. For example, the presence of greater changes observable in gray matter volume could reflect a contribution of changes in water content across the menstrual cycle rather than changes in neuronal and glial structures within the gray matter. While volumetric and cortical thickness estimates are both derived from T1w magnetic resonance imaging (MRI) data, water content variations are more likely to affect volumetric measures due to shifts in extracellular fluid dynamics, which may be influenced by hormonal changes^56–58^, than cortical thickness measures, which are less sensitive to such transient changes^59^.

Preclinical literature indicates that progesterone exerts an inhibitory effect on proliferative actions of estradiol^5^. For example, animal studies have shown that estradiol enhances the excitability of fast-spiking interneurons in deep cortical layers^60^ and increases synapse formation in the prefrontal cortex^8^. However, concurrent cyclic administration of progesterone attenuates this increase in spine density when paired with estradiol^61^. Additionally, progesterone exhibits a similar inhibitory effect on dendritic spines in the hippocampus^10^. In line with these findings, our study suggests that individuals with typical menstrual cycles exhibit a heightened sensitivity to progesterone. We observed fluctuations in brain volumes over the monthly period in both typical cycles and in the case of hormonal dysregulation, with progesterone exerting a more pronounced influence on structural brain dynamics in typical cycles. These findings are consistent with previous research using the 28andMe dataset, revealing substantial associations between progesterone and the medial temporal lobe. These associations were abolished when progesterone was selectively suppressed and estradiol dominated^40^. In contrast, when estradiol is the dominating hormone throughout the cycle, as observed in endometriosis, it appears to exert a greater impact on structural brain dynamics, potentially exerting its proliferative actions. Our findings align with previous literature^48–50,52^, indicating elevated estradiol levels and estradiol dominance in the luteal phase of the menstrual cycle in endometriosis, suggesting a greater exposure of estradiol on the brain. Additionally, our results in the female using OC, providing an additional dataset with a hormonal milieu similar to the endometriosis cycle, further underscore the influence of estradiol dominance on brain structure. Voxel-wise analyses further supported these associations. While implicated regions varied between individuals, the most consistent finding, across both voxel-wise and SVD analyses, was that progesterone was the primary correlate of brain volume changes in the typical cycles, whereas estradiol was the primary correlate in the endometriosis and OC cycle.

Estrogen is believed to have a neuroprotective role, promoting brain health and protecting against cognitive decline^62–64^. However, while estradiol levels within the physiological range stimulate brain activity, especially in the hippocampus, supraphysiological levels of estradiol (equivalent to those during early pregnancy) exhibit opposite effects^65^. Interestingly, unopposed estrogen during hormone replacement therapy in menopause enhances activation of fronto-cingulate regions during cognitive functioning tasks^66^. This highlights the specific impact of elevated estrogen levels, unbalanced by other hormones, on brain activity and cognition. Little is known about the impact of prolonged high estradiol exposure during the reproductive years on long-term health outcomes. This underscores the importance of further research to elucidate the longitudinal relationships among gonadal hormones, reproductive health and long-term well-being in individuals with hormonal dysregulations.

To further contextualize our findings, we expanded the scope of our study by including additional analyses of one male over a densely sampled 5-week period. While VSTPs fluctuated over the 5-week period, these changes were not associated with hormone concentrations. This is not surprising given that the substantially reduced magnitude of hormonal fluctuations in the male participant compared to what is observed and characteristic of a menstrual cycle. It also suggests that the observed spatiotemporal fluctuations may not be detectably driven by those hormones but could be influenced by factors not accounted for in this investigation, such as intake of water, or cerebral blood flow. Furthermore, these results may indicate the presence of different regulatory mechanisms or hormonal thresholds in males compared to females. However, this requires further investigation in future studies that explore diurnal changes or manipulate hormones in males. Such studies can provide clearer insights into sex and sex-hormone differences as most recently demonstrated^67^. Furthermore, the absence of substantial hormone–brain associations in the male participant suggests that the associations observed in female participants are likely driven by cyclical variations in gonadal hormones rather than general intersession variability and underscores the importance of studying female-specific endocrinological influences on brain structure. This area of research has historically been underrepresented in the field of neuroscience.

The study has several limitations. First, because these are dense-sampling datasets with a limited sample size, caution is advised when generalizing the findings to the broader population. By focusing on individual participants, we aimed to mitigate the intra-individual variability of hormonal and brain structural fluctuations, thereby providing clearer insight into personalized spatiotemporal patterns that are often obscured in studies with larger samples. Our approach provides a more precise examination of the specific patterns of brain structure and hormonal fluctuations at an individual level, offering a higher level of sensitivity and temporal resolution toward precision imaging. Second, this study applied a model-free whole-brain approach. Using SVD represents a new method for exploring short-term structural brain changes across the menstrual cycle. This approach helps to identify unique spatiotemporal profiles, thereby potentially mechanistic principles underlying structural brain changes throughout the menstrual cycle. The data-driven nature of our approach contrasts with the more common hypothesis-driven studies that focus on predefined regions of interest. While our model-free strategy allows for the discovery of hormone–brain associations in less commonly studied areas, it did not identify particular regions consistently across individuals to target in future research. Instead, it highlights that the entire brain undergoes individual structural changes across menstrual cycles, changes that are partly driven by gonadal hormones. However, all imaging data used in these analyses will be made openly available upon publication, allowing for targeted follow-up analyses using regions of interest or established network templates. Third, we identified unique temporal patterns in each participant, precluding direct comparisons between them. Moreover, variations in sampling strategies were observed among participants. While the 28andMe (typical) cycle was sampled daily for four consecutive weeks, scanning in the typical, the endometriosis and the OC cycle occurred primarily on weekdays for five consecutive weeks. For instance, the longest scanning gap in the typical cycle spanned 4 days. These differences might explain why weaker associations were observed in the typical cycle and stronger associations in the 28andMe (typical) cycle. Variations in scanning schedules and differences in participants’ age and factors such as nicotine use in one participant may contribute to divergent temporal patterns that should not be directly compared. For instance, nicotine acutely inhibits aromatase in the thalamus in healthy females, thereby it blocks the local synthesis of estrogen from androgen precursors^68^. Notably, the finding that estradiol levels were associated with brain volume in estradiol-dominant cycles and progesterone levels in progesterone-dominant cycles was more consistent than the specific regions implicated, suggesting robust yet individualized brain–hormone coupling. These results underscore the need to focus on personalized spatiotemporal patterns in both brain structure and hormonal levels. Menstrual cycle dynamics and other intra-individual factors that influence our measures of interest are inherently variable within-person^69^. Thus, while there is some consistency across individuals and cycles in the dominant spatiotemporal pattern and the voxel-wise analysis (precision), hormone–brain associations remain noisy and difficult to replicate across individuals. Fourth, our study revealed dynamic brain changes not only in females but also in a male participant. In females, these changes were associated with fluctuations in estradiol and progesterone levels, but the mechanisms driving similar changes in males remain unclear. Finally, we compared gonadal hormone levels among the four participants, but different steroid analyses were used in the typical, the endometriosis and the OC cycle compared to the 28andMe (typical) cycle. Hormones were identified through immunoassay (IAs) in the typical, endometriosis and the OC cycle, while, in the 28andMe (typical) cycle, hormones were identified through liquid chromatography–mass spectrometry (LC–MS). While IAs offer a higher sample turnover, they are limited in trueness, precision and sensitivity. In contrast, LC–MS has been demonstrated to deliver better sensitivity and specificity. However, good overall method agreement was found for estradiol and progesterone^70,71^. Future studies should consider using consistent steroid analyses to ensure comparability, or harmonization methods should be developed to enable the integration of hormone assessments, allowing the pooling of data from multiple research sites to increase power, reproducibility and generalizability^72^.

Further research using whole-brain approaches and spatiotemporal patterns with larger and more diverse samples is necessary to validate and expand these initial findings. Future research should address potential interindividual variations and strive to enhance the generalizability of the observed associations. Despite the small sample size, our findings provide valuable initial insights into the dynamic impact of hormonal fluctuations on whole-brain structural plasticity throughout the menstrual cycle and under conditions of nontypical hormonal regulation. While specific regional changes were not the focus of this study, the consistent spatial maps and unique temporal patterns emphasize a widespread, coordinated influence of hormonal changes on brain structure. From a translational perspective, our findings hold important implications for the interpretation of animal studies on hormone–brain interactions. While animal models provide valuable insights into cellular and molecular mechanisms, our results emphasize that hormone-driven volumetric changes in humans are not confined to limbic structures, such as the hippocampus, but extend to widespread cortical and cerebellar regions. Future studies should aim to integrate methodologies that allow for cross-species comparisons, ensuring that findings from animal models align with the distributed brain networks implicated in human neuroendocrine dynamics. Furthermore, animal models of hormone–brain interactions often focus on acute manipulations of estradiol or progesterone. Yet, our data emphasize the importance of naturally occurring hormone fluctuations and their interaction over time. Given the distinct patterns observed in cycles with estradiol dominance versus typical cycles, future animal studies should consider the broader hormonal milieu rather than focusing on individual hormones in isolation.

In summary, our study lays the groundwork for a future in personalized and precision medicine, offering initial insights into how distinct hormonal milieus—such as the interplay between estradiol and progesterone levels in typical cycles or estradiol dominance in endometriosis—affect brain structure. Rather than identifying brain regions universally linked to specific hormones, our results underscore that hormone–brain associations vary across individuals and are milieu-dependent. These associations appear to be influenced by the presence or the absence of natural hormonal fluctuations, emphasizing the importance of within-person designs to capture the dynamic nature of hormone-related brain plasticity.

## Methods

Dense sampling, longitudinal datasets were acquired from three female participants in Jena, Germany. These datasets are referred to as the ‘endometriosis cycle’, ‘typical cycle’ and the ‘OC cycle’. To extend our findings, we also leveraged the open-access 28andMe dataset of one female, which probes the extent to which endogenous fluctuations in sex hormones across a complete reproductive cycle influence the brain^33–38,40^. The data were acquired in Santa Barbara, California, and are referred to as ‘28andMe (typical) cycle’.

For the purposes of control analyses and to probe comparability of our findings, an additional dense sampling, longitudinal dataset of one male was acquired over the time course of 5 weeks in Jena, Germany.

All participants (*n* = 5) gave written informed consent. The Friedrich Schiller University Jena Ethics Committee (for participants acquired in Jena) and the University of California, Santa Barbara Human Subjects Committee (for participants acquired in Santa Barbara) approved the study. Participants were not compensated. All imaging data are openly available.

### Participants

#### Primary analyses

The study procedures for the participants in Jena, Germany, were as follows: the first healthy female (37 years of age, Caucasian) underwent most weekday testing for five consecutive weeks (9 January–12 February 2023) while freely cycling, resulting in 25 test sessions. The female participant (‘typical cycle’) had a history of regular menstrual cycles (last half-year mean length = 27.1 days, s.d. = 0.64, range = 26–28 days), no history of psychiatric, neurological and endocrine diagnoses, breastfeeding or pregnancy, and no history of alcohol or drug abuse, but the current use of nicotine. The second female participant (30 years of age, Caucasian) diagnosed with endometriosis (‘endometriosis cycle’) participated in this dense sampling, longitudinal study. She received the diagnosis 7 months before the assessments (28 October 2022) after a cyst surgery in the pelvic area. The participant was tracking her menstrual cycle length and reported a mean menstrual cycle length of 24.4 days (s.d. = 1.67, range = 23–27 days) during that time. Otherwise, the female participant had no history of psychiatric or neurological disorders, breastfeeding or pregnancy, and no history of smoking, alcohol or drug abuse. The participant underwent testing from Monday to Friday for five consecutive weeks (12 June–14 July 2023) while freely cycling, resulting in 25 test sessions. The third healthy female (31 years of age, Caucasian) underwent most weekday testing for five consecutive weeks (27 March–28 April 2023), resulting in 25 test sessions. Before the assessments, the participant had been prescribed a combined OC pill (0.03-mg ethinyl-estradiol, 2-mg dienogest, Maxim, Jenapharm) approximately 3 months before study initiation. The female participant (‘OC cycle’) had no history of psychiatric, neurological or endocrine diagnoses, nor had she experienced breastfeeding or pregnancy. Furthermore, she had no history of alcohol or drug abuse and did not use nicotine.

The study procedure for the fourth participant was as follows: a healthy female participant (23 years of age, Caucasian, ‘28andMe (typical) cycle’) underwent testing for 30 consecutive days (9 July–7 August 2018) while freely cycling. She had a history of regular menstrual cycles (no missed periods, cycle occurring every 26–28 days) and had not taken hormone-based medication in the 12 months before the first study. The participant had no history of psychiatric or neurological disorders, breastfeeding or pregnancy, and no history of smoking, alcohol or drug abuse.

#### Additional analyses (male participant)

The fifth participant, a healthy male (36 years of age, Caucasian), underwent most weekday testing for five consecutive weeks (4 May–7 June 2023), resulting in 25 test sessions. The male participant (‘male’) had no history of psychiatric, neurological or endocrine diagnoses, and reported no instances of alcohol, drug or nicotine abuse.

#### Image acquisition

For datasets collected in Jena (typical cycle, endometriosis cycle, OC, male), scans were collected at 7.30 a.m. (±30 min) local time. The imaging dataset for the typical cycle was acquired on a 3 T MRI scanner (Prisma, Siemens Medical Solutions; software version MR E11) with a 64-channel head coil. The imaging datasets for the endometriosis cycle, male and female on OC, were acquired on a 3T MRI scanner (Prisma, Siemens Medical Solutions; software version MR XA30) with a 64-channel head coil. Structural MRI for the datasets was acquired with T1w magnetization prepared–rapid gradient echo sequence with the generalized autocalibrating partially parallel acquisitions acceleration. Scan parameters were as follows: echo time = 2.22 ms, repetition time = 2,400 ms, inversion time = 1,000 ms, flip angle = 8°, matrix size = 320 × 320 pixels, field of view = 256 mm, band width = 220 Hz pixel^−1^ and slice thickness = 0.80 mm.

For the 28andMe (typical) cycle dataset, scans were collected on a 3 T MRI scanner (Prisma, Siemens Medical Solutions; software version MR D13D) equipped with a 64-channel head coil. Structural scans were acquired using a T1w magnetization prepared–rapid gradient echo sequence with the generalized autocalibrating partially parallel acquisitions acceleration with the following parameters: echo time = 2.31 ms, repetition time = 2,500 ms, inversion time = 934 ms, flip angle = 7°, matrix size = 320 × 320 pixels, field of view = 255 mm, band width = 210 Hz pixel^−1^ and slice thickness = 0.80 mm.

#### Image preprocessing

The parameters used to acquire the images (for example, sizes, space directions and space origin) and the quality of the images (for example, motion artifacts, ringing, ghosting of the skull or eyeballs, cutoffs, signal drops and other artifacts) were visually inspected. One scan from the endometriosis cycle (test day 8) had to be removed due to artifacts in subcortical structures, corpus callosum and cingulate gyrus (measurements from this test day were excluded for all statistical analyses). The final datasets consisted of 24 T1w images for the endometriosis cycle, 25 T1w images for the typical cycle, 25 T1w images for the OC cycle, 30 T1w images for the 28andMe (typical) cycle and 25 T1w images for the male.

The T1w images were converted from DICOM to NIfTI files using dcm2niix (version v1.0.20170724, https://www.nitrc.org/projects/mricrogl/) and then preprocessed in SPM12 (version r7771, http://www.fil.ion.ucl.ac.uk/spm) and the CAT12 (version 12.9, https://neuro-jena.github.io/cat)^55^ toolbox using the (plasticity) longitudinal pipeline approach in Matlab (The MathWorks, version R2021b). All T1w images were corrected for bias-field inhomogeneities and initially tissue-classified into gray matter, white matter and cerebrospinal fluid^73^, followed by an adaptive maximum a posteriori segmentation^74^, which also accounts for partial volume effects^75^. The resulting gray and white matter partitions were spatially normalized to MNI space, Geodesic Shooting Registration^76^. Subsequently, the normalized tissue segments were smoothed using a 6-mm full-width at half-maximum Gaussian Kernel. The extraction of cortical surfaces uses a projection-based thickness method^77^ to estimate initial cortical thickness and central surface simultaneously. Topological defects are corrected using spherical harmonics^78^, followed by surface refinement to produce final central, pial and white surface meshes. These surfaces refine the initial thickness measurement using the FreeSurfer metric^79^. Subsequently, the individual central surfaces are aligned to the FreeSurfer FsAverage template hemisphere, spherically inflated to minimize distortions^80^ and spherically registered using a two-dimensional DARTEL approach^81,82^.

#### Image quality and motion assessment

We conducted a quality assessment of all T1w images using the Image Quality Rating tool (https://neuro-jena.github.io/cat12-help/). Image quality was evaluated based on assigned values, with ratings of 1 and 2 indicating (very) good image quality (grades A and B), while values around 5 and higher suggest problematic image quality (grades E and above). Notably, all assessed images exhibited excellent to good quality (endometriosis cycle—*M* = 1.407, s.d. = 0.002; typical cycle—*M* = 1.471, s.d. = 0.002; 28andMe (typical) cycle—*M* = 1.480, s.d. = 0.002; OC cycle—*M* = 1.503, s.d. = 0.003; male—*M* = 1.469, s.d. < 0.001).

Furthermore, mean framewise displacement (FWD), derived from a 12-min resting-state functional scan acquired before the T1w scans, was extracted to indicate motion across the entire scan duration (approximately 55 min). The MRI protocol included a resting-state functional scan for all participants, except for the typical cycle (here the functional scan was replaced with a magnetic resonance spectroscopy scan). Mean FWD was extremely minimal across all participants (endometriosis cycle—*M* = 0.121 mm, s.d. = 0.009 mm; OC cycle—*M* = 0.098 mm, s.d. = 0.009 mm; male—*M* = 0.137 mm, s.d. = 0.011 mm; Supplementary Fig. 1). Mean FWD for the 28andMe (typical) cycle is found elsewhere^40^ and did not exceed 0.150 mm.

#### Endocrine procedure

For the datasets acquired in Jena, Germany, a blood draw was immediately followed by the MRI session at 8:30 a.m. (±30 min). One 4.9-ml blood sample was collected in an S-Monovette Serum-GEL (Sarstedt) with a clotting activator/gel at each test session. The sample was clotted at room temperature and centrifuged (2,500*g* for 10 min) within 2 h. Estradiol (pmol l^−1^), luteinizing hormone (LH; IU l^−1^), follicle-stimulating hormone (FSH; IU l^−1^) and progesterone serum concentrations (nmol l^−1^) were determined at the Institute of Clinical Chemistry and Laboratory Diagnostics, Jena University Hospital. Estradiol was assessed with the electrochemiluminescence immunoassay (ECLIA) Elecsys Estradiol III Assay. Assay antibodies, measuring ranges (defined by the limit of detection and the maximum of the master curve) and intra-assay precision coefficients of variation for estradiol were as follows: antibodies, two biotinylated monoclonal anti-estradiol antibodies (rabbit), 2.5 ng ml^−1^ and 4.5 ng ml^−1^; measuring range, 18.4–11,010 pmol l^−1^ (5–3,000 pg ml^−1^); intra-assay precision, ≤8.4% variation coefficient. LH was assessed with the ECLIA Elecsys LH Assay. Assay antibodies, measuring ranges and intra-assay coefficients of variation for LH were as follows: antibodies, biotinylated monoclonal anti-LH antibody (mice), 2.0 mg l^−1^; measuring range, 0.3–200 mIU ml^−1^ (0.3–200 IU l^−1^); intra-assay precision, ≤2.2% variation coefficient. FSH was assessed with the ECLIA Elecsys FSH Assay. Assay antibodies, measuring ranges and intra-assay coefficients of variation for FSH were as follows: antibodies, biotinylated monoclonal anti-FSH antibody (mice), 0.5 mg l^−1^; measuring range, 0.3–200 mIU ml^−1^ (0.3–200 IU l^−1^); intra-assay precision, ≤2.1% variation coefficient. Progesterone was assessed with the ECLIA Elecsys Progesterone III Assay. Assay antibodies, measuring ranges and intra-assay coefficients of variation for progesterone were as follows: antibodies, biotinylated monoclonal antiprogesterone antibody (recombinant sheep), 30 ng ml^−1^; measuring range, 0.159–191 nmol l^−1^ (0.05–60 ng ml^−1^); intra-assay precision, ≤20.7% variation coefficient. All assays were determined on the cobas e 402/801 analyzer (Roche Diagnostics GmbH) and were used according to the manufacturer’s instructions. The reported intra-assay precision and coefficient of variation values are taken from the manufacturer’s package inserts and reflect the analytical performance of the assays. These values are based on Roche’s validation studies and do not represent quality control data generated at the Institute of Clinical Chemistry and Laboratory Diagnostics, Jena University Hospital, Jena, Germany.

For the 28andMe (typical) cycle dataset acquired in Santa Barbara, CA, USA, a licensed phlebotomist inserted a saline-lock intravenous line into the dominant or nondominant hand or forearm. One 10-ml blood sample was collected in a vacutainer SST (BD Diagnostic Systems) each session. The sample was clotted at room temperature for 45 min until centrifugation (2,000*g* for 10 min) and then aliquoted into three 1-ml microtubes. Serum samples were stored at −20 °C until assayed. Serum concentrations were determined at the Brigham and Women’s Hospital Research Assay Core. Estradiol and progesterone were assessed through LC–MS. Assay sensitivities, dynamic range and intra-assay coefficients of variation (respectively) were as follows: estradiol—1 pg ml^−1^, 1–500 pg ml^−1^, <5% relative s.d.; progesterone—0.05 ng ml^−1^, 0.05–10 ng ml^−1^, 9.33% relative s.d. FSH and LH levels were determined using chemiluminescent assay (Beckman Coulter). The assay sensitivity, dynamic range and intra-assay coefficient of variation were as follows: FSH—0.2 mIU ml^−1^, 0.2–200 mIU ml^−1^, 3.1–4.3%; LH—0.2 mIU ml^−1^, 0.2–250 mIU ml^−1^, 4.3–6.4%.

#### Analysis approach

Please note that measurements from test day 8 of the endometriosis cycle were excluded from all statistical analyses to ensure consistency in the number of test days across all analyses.

#### Hormone concentrations

Statistical analyses of hormone concentrations were performed using Statistical Package for Social Sciences (SPSS; version 27). First, a one-way multivariate analysis of variance was conducted with estradiol levels, progesterone levels and progesterone-to-estradiol ratio as dependent variables. The fixed factors were the four individuals (endometriosis cycle, OC cycle, typical cycle and 28andMe (typical) cycle). Post hoc analyses of variance and two-tailed *t*-tests were performed and Bonferroni-corrected.

#### Structural brain measures

First, SVD was used to extract spatiotemporal patterns from the preprocessed images by decomposing the three-dimensional image sets into spatial patterns (spatial component) and their associated temporal dynamics (time course and temporal component). The spatial patterns represent the brain regions that share similar spatial changes, while the temporal component reflects these changes evolve over time. To ensure consistency in spatial patterns while allowing for distinct temporal patterns, the typical cycle, the 28andMe (typical) cycle, the endometriosis cycle and the OC cycle were modeled together by concatenating the data from these participants. For the male participants, who do not have a menstrual cycle, the SVD was performed separately to account for the unique dynamics.

By using SVD, we can identify and analyze these patterns, revealing coherent time courses across the brain rather than being restricted to an expected change over time. This approach is analogous to applying independent component analysis to resting-state functional MRI data. However, while the motivation here is to identify underlying independent processes or networks, the objective of our study was to decompose the structural data into orthogonal (nonoverlapping) components. Furthermore, SVD provides consistent and repeatable patterns, which are crucial for reproducibility of the results across different datasets.

Using a flexible modeling approach, we assessed the variations in whole-brain volumetric and CSTPs across the monthly period. Specifically, we used a GAM using the ‘mgcv’ package (version 1.9–1) in RStudio (version 2024.04.1 + 748), which allows the independent variable (test days) to influence the outcome through smooth, nonlinear functions, to address potential nonlinear effects in volumetric and cortical thickness brain dynamics. The default value of *k* = 10 was used to determine the smoothness of the functions. This approach acknowledges the anticipated complexity and nonlinearity of the relationship between the menstrual cycle and brain structure, enabling a more adaptable modeling of menstrual cycle-dependent trajectories in structural brain dynamics. Initially, we also considered models with autoregressive terms to account for potential temporal dependencies in the data. However, model checks indicated that including autoregressive terms led to overfitting. There, we opted for the simpler GAM model, which provided a more reliable and interpretable fit. The following GAMs were fitted for the VSTPs for each individual separately:

$${\rm{VSTP}}1={\beta }_{0}+{f}_{1}({\rm{test}}\,{\rm{day}})$$

$${\rm{VSTP}}2={\beta }_{0}+{f}_{1}({\rm{test}}\,{\rm{day}})$$

$${\rm{VSTP}}3={\beta }_{0}+{f}_{1}({\rm{test}}\,{\rm{day}})$$

The following GAMs were fitted for the CSTPs for each individual separately (CSTP3 in the male only):

$${\rm{CSTP}}1={\beta }_{0}+{f}_{1}({\rm{test}}\,{\rm{day}})$$

$${\rm{CSTP}}2={\beta }_{0}+{f}_{1}({\rm{test}}\,{\rm{day}})$$

$${\rm{CSTP}}3={\beta }_{0}+{f}_{1}({\rm{test}}\,{\rm{day}})$$

GAMs were adjusted for multiple comparisons using the FDR method^83^.

Next, we assessed the relationship between the dynamics of volumetric and cortical thickness and gonadal hormones. To stabilize variances, gonadal hormone levels were transformed using the square root. We then used time-series regression models with VSTPs and CSTPs as dependent variables and the gonadal hormones as predictors in SPSS (version 27). These models captured the relationship between current structural dynamics and current gonadal hormone concentrations. The following time-series regression models were fitted for the VSTP1 for each individual separately:

$${\rm{VSTP}}1={\beta }_{0}+{\beta }_{1}\times {\rm{estradiol}}+\varepsilon$$

$${\rm{VSTP}}1={\beta }_{0}+{\beta }_{1}\times {\rm{progesterone}}+\varepsilon$$

$${\rm{VSTP}}1={\beta }_{0}+{\beta }_{1}\times {\rm{ratio}}+\varepsilon$$

The same time-series regression models were fitted for VSTP2 and VSTP3, as well as CSTP1 and CSTP2 for each individual separately (CSTP3 in the male only). Additionally, we explored functional regression analyses incorporating autoregressive terms to account for potential dependencies in the data. However, model checks indicated that including autoregressive terms resulted in overfitting. As a result, we decided to use the initial simpler model without these terms. Finally, because not all variables were normally distributed, relationships were further investigated using nonparametric Spearman rank correlation, as implemented in the ‘stats’ package (version 4.4.0) in RStudio (version 2024.04.1 + 748). Results were highly consistent across both approaches. All models were adjusted for multiple comparisons using the FDR method^83^.

Finally, to investigate the association between hormonal concentrations and structural brain measures at each voxel or vertex, we performed a statistical analysis using a general linear model in CAT12. Hormonal concentrations were included as the dependent variable in a regression framework. To identify statistically significant effects, we used the threshold-free cluster enhancement method (https://neuro-jena.github.io/software.html#tfce)^84^, which integrates both the magnitude and spatial extent of effects and controls for multiple comparisons by applying a family-wise error correction with a significance threshold of *P* < 0.01. For voxel-wise analyses, voxels with an absolute threshold below 0.1 were excluded to focus exclusively on gray matter regions.

### Reporting summary

Further information on research design is available in the Nature Portfolio Reporting Summary linked to this article.

## Online content

Any methods, additional references, Nature Portfolio reporting summaries, source data, extended data, supplementary information, acknowledgements, peer review information; details of author contributions and competing interests; and statements of data and code availability are available at 10.1038/s41593-025-02066-2.

## Supplementary information

Supplementary Information Supplementary Tables 1–11 and Supplementary Fig. 1.

Reporting Summary

## Source data

Source Data Fig. 1 Raw hormonal levels, statistical source data for female participants (*n* = 4).

Source Data Fig. 3 Component weights of SVD from volumetric spatial patterns and cortical thickness spatial patterns (*n* = 4).

Source Data Fig. 4 Square-rooted and standardized hormonal levels, standardized eigenvalues for volumetric spatiotemporal patterns, statistical source data for female participants (*n* = 4).

Source Data Fig. 5 Square-rooted and standardized hormonal levels, standardized eigenvalues for cortical thickness spatiotemporal patterns, statistical source data for female participants (*n* = 4).

Source Data Fig. 6 Statistical source data from GLM (voxel-based; *n* = 4).

Source Data Fig. 7 Statistical source data from GLM (vertex-based; *n* = 4).

Source Data Extended Data Fig. 2 Raw hormonal levels for male participant (*n* = 1).

Source Data Extended Data Fig. 3 Square-rooted and standardized hormonal levels, Standardized eigenvalues for volumetric spatiotemporal patterns, Statistical source data for male participant (*n* = 1).

Source Data Extended Data Fig. 4 Square-rooted and standardized hormonal levels, Standardized eigenvalues for cortical thickness spatiotemporal patterns, Statistical source data for male participant (*n* = 1).

Source Data Extended Data Fig. 5 Statistical source data from GLM in the male (voxel-based and vertex-based; *n* = 1).

## Data Availability

The datasets generated in Jena, Germany, are available at https://openneuro.org/datasets/ds006491. The dataset generated in Santa Barbara, CA, USA, is available at https://openneuro.org/datasets/ds002674. Source data are provided with this paper.

## Code availability

Code is available in a public repository and can be found online at https://github.com/ChristianGaser/menstrual-brain-structural-dynamics.
