# Publisher Correction: Hormonal milieu influences whole-brain structural dynamics across the menstrual cycle using dense sampling in multiple individuals

**DOI:** 10.1038/s41593-025-02163-2

**Published:** 2025-11-25

**Authors:** Carina Heller, Daniel Güllmar, Lejla Colic, Laura Pritschet, Martin Gell, Nooshin Javaheripour, Feliberto de la Cruz, Philine Rojczyk, Carina J. Koeppel, Bart Larsen, Habib Ganjgahi, Frederik J. Lange, Ann-Christine Buck, Tim L. Jesgarzewsky, Robert Dahnke, Michael Kiehntopf, Emily G. Jacobs, Zora Kikinis, Martin Walter, Ilona Croy, Christian Gaser

**Affiliations:** 1 Department of Psychiatry and Psychotherapy, Jena University Hospital, Jena, Germany; 2 Masonic Institute for the Developing Brain, University of Minnesota, Minneapolis, MN USA; 3 Department of Pediatrics, University of Minnesota, Minneapolis, MN USA; 4 Department of Psychological and Brain Sciences, University of California, Santa Barbara, Santa Barbara, CA USA; 5 German Center for Mental Health (DZPG), Jena–Magdeburg–Halle, Germany; 6 Center for Intervention and Research on Adaptive and Maladaptive Brain Circuits Underlying Mental Health (C-I-R-C), Jena–Magdeburg–Halle, Germany; 7 Medical Physics Group, Institute of Diagnostic and Interventional Radiology, Jena University Hospital, Jena, Germany; 8 Department of Psychiatry, University of Pennsylvania, Philadelphia, PA USA; 9 Department of Psychiatry and Behavioral Sciences, University of Minnesota, Minneapolis, MN USA; 10 Institute of Neuroscience and Medicine (INM-7: Brain & Behaviour), Research Centre Jülich, Jülich, Germany; 11 Lab for Autonomic Neuroscience, Imaging and Cognition (LANIC), Department of Psychosomatic Medicine and Psychotherapy, Jena University Hospital, Jena, Germany; 12 cBRAIN, Department of Child and Adolescent Psychiatry, Psychosomatics, and Psychotherapy, Ludwig-Maximilians-University, Munich, Germany; 13 Psychiatry Neuroimaging Laboratory, Department of Psychiatry, Brigham and Women’s Hospital, Harvard Medical School, Boston, MA USA; 14 Department of Psychiatry and Neurosciences, Charité – Universitätsmedizin Berlin, corporate member of Freie Universität Berlin and Humboldt-Universität zu Berlin, Berlin, Germany; 15 Institute of Child Development, Univeristy of Minnesota, Minneapolis, MN USA; 16 Big Data Institute, Li Ka Shing Centre for Health Information and Discovery, Nuffield Department of Population Health, University of Oxford, Oxford, UK; 17 Department of Statistics, University of Oxford, Oxford, UK; 18 Oxford University Centre for Integrative Neuroimaging, FMRIB, Nuffield Department of Clinical Neurosciences, University of Oxford, Oxford, UK; 19 Department of Clinical Psychology, Friedrich Schiller University Jena, Jena, Germany; 20 Department of Neurology, Jena University Hospital, Jena, Germany; 21 Institute of Clinical Chemistry and Laboratory Diagnostics, Jena University Hospital, Jena, Germany; 22 Neuroscience Research Institute, University of California, Santa Barbara, Santa Barbara, CA USA; 23 Department of Psychotherapy and Psychosomatic Medicine, University Hospital Carl Gustav Carus, Technische Universität Dresden, Dresden, Germany

**Keywords:** Neuroscience, Brain, Neuroendocrine diseases

Correction to: *Nature Neuroscience* 10.1038/s41593-025-02066-2, published online 26 September 2025.

In the version of the article initially published, in Fig. 1a, the y-axis units were “pmol ml^−1^” and “nmol ml^−1^” and have now been corrected to “pmol l^−1^” and “nmol l^−1^”. Throughout the text, figures and Supplementary table footnotes, the “progesterone-to-estradiol” ratio was originally written “estradiol-to- progesterone.” The text, figures and Supplementary information have been amended in the HTML and PDF versions of the article.

