# Supplementary material for: Hormonal milieu influences whole-brain structural dynamics across the menstrual cycle using dense sampling in multiple individuals
Source: Nat Neurosci. 2025 Sep 26;28(12):2588–600. doi: 10.1038/s41593-025-02066-2 (PMC12672370; doi:10.1038/s41593-025-02066-2)
Supplement: Supplementary file 1 — Supplementary Tables 1–11 and Supplementary Fig. 1. [file 41593_2025_2066_MOESM1_ESM.pdf]

nature neuroscience

Supplementary information

https://doi.org/10.1038/s41593-025-02066-2

Hormonal milieu influences whole-brain structural dynamics across the menstrual cycle using dense sampling in multiple individuals

In the format provided by the authors and unedited

Supplementary Table 1. Largest significant clusters in volumetric spatiotemporal patterns across the endometriosis cycle, the oral contraceptives cycle, the typical cycle, and the 28andMe (typical) cycle.

Region	VSTP1	VSTP2	VSTP3
**Angular gyrus**	3475	186	---
**Anterior cingulate gyrus**	---	517	937
**Anterior insula**	---	234	184
**Calcarine cortex**	---	929	397
**Cerebellum**	19758	18432	19659
**Cuneus**	---	959	397
**Entorhinal area**	---	436	101
**Frontal pole**	---	300	937
**Fusiform gyrus**	2294	1239	836
**Gyrus rectus**	---	1139	741
**Hippocampus**	---	310	---
**Inferior occipital gyrus**	1147	856	---
**Inferior temporal gyrus**	3442	1687	136
**Lingual gyrus**	4589	1018	826
**Medial frontal cortex**	---	550	485
**Middle cingulate gyrus**	166	338	937
**Middle frontal gyrus**	5736	548	3589
**Middle occipital gyrus**	---	181	---
**Middle temporal gyrus**	5736	1587	104
**Occipital pole**	---	380	---
**Operculum**	1147	283	729
**Orbital gyrus**	---	864	341
**Parietal operculum**	---	---	256
**Planum temporale**	---	169	182
**Postcentral gyrus**	1147	642	322
**Posterior cingulate gyrus**	---	---	314
**Precentral gyrus**	2294	1393	1234
**Precuneus**	6883	991	1391
**Putamen**	---	584	810
**Subcallosal area**	---	275	256
**Superior frontal gyrus**	2294	922	6006
**Superior occipital gyrus**	---	512	---
**Superior parietal lobule**	2294	414	---
**Superior temporal gyrus**	3442	1168	162
**Supplementary motor cortex**	---	1079	1874
**Supramarginal gyrus**	1310	348	---
**Temporal pole**	1254	695	219
**Thalamus**	2294	2596	1129
**Transverse temporal gyrus**	---	223	---

Note. Singular Value Decomposition (SVD) analysis was employed to extract volumetric spatiotemporal patterns (VSTP). SVD decomposed the images into spatiotemporal components, reflecting patterns of brain structure over time. To capture shared spatial patterns across individuals, data from all cycles were concatenated. Only clusters with ≥ 100 voxels are reported. Largest clusters are indicated in purple. VSPT1 = volumetric spatiotemporal pattern 1. VSPT2 = volumetric spatiotemporal pattern 2. VSPT3 = volumetric spatiotemporal pattern 3.

Supplementary Table 2. Results of Generalized Additive Models (GAMs) in volumetric spatiotemporal patterns.

Cycle	Term	Outcome	edf	*F*	*p*	p_{FDR}	R^2	*Deviance explained*
**Endometriosis**	**s(Day)**	**VSTP1**	8.819	194.8	***<0.001***	***<0.001***	0.987	99.2%
		**VSTP2**	8.687	34.7	***<0.001***	***<0.001***	0.931	95.7%
		**VSTP3**	8.635	44.2	***<0.001***	***<0.001***	0.945	96.6%
**Oral Contraceptives**	**s(Day)**	**VSTP1**	8.788	351.5	***<0.001***	***<0.001***	0.992	99.5%
		**VSTP2**	7.748	145.5	***<0.001***	***<0.001***	0.981	98.7%
		**VSTP3**	8.064	50.9	***<0.001***	***<0.001***	0.949	96.6%
**Typical Cycle**	**s(Day)**	**VSTP1**	8.217	205.7	***<0.001***	***<0.001***	0.987	99.1%
		**VSTP2**	8.617	35.0	***<0.001***	***<0.001***	0.928	95.4%
		**VSTP3**	8.630	207.8	***<0.001***	***<0.001***	0.987	99.2%
**28andMe (Typical) Cycle**	**s(Day)**	**VSTP1**	7.909	108.0	***<0.001***	***<0.001***	0.970	97.8%
		**VSTP2**	8.629	672.1	***<0.001***	***<0.001***	0.995	99.7%
		**VSTP3**	7.380	11.0	***<0.001***	***<0.001***	0.758	81.9%

Note. GAMs were performed to assess whether volumetric spatiotemporal patterns (VSTP1, VSTP2, VSTP3) fluctuated across the monthly period. The term s(Day) represents a smooth function of Day, modeling potential nonlinear fluctuations over time. The effective degrees of freedom (edf) indicate the complexity of the smooth term, with higher values suggesting greater flexibility. p_{FDR} = *p*-values after False Discovery Rate (FDR) correction for multiple comparisons. Significant results are indicated in bold.

Supplementary Table 3. Results of linear regressions and Spearman correlations in volumetric spatiotemporal patterns.

			Linear regressions							Spearman correlations		
**Cycle**	**Outcome**	**Predictor**	**Estimate β**	***SE***	***T***	***p***	p_{FDR}	R^2	***RMSE***	***rho***	***p***	p_{FDR}
**Endometriosis**	**VSTP1**	**Estradiol**	0.006	0.002	3.415	***0.002***	***0.010***	0.346	0.088	0.571	***0.004***	***0.037***
		**Progesterone**	0.015	0.012	1.287	0.212	0.424	0.070	0.105	0.264	0.211	0.399
		**Ratio**	0.008	0.010	0.816	0.423	0.635	0.029	0.107	0.016	0.943	0.943
	**VSTP2**	**Estradiol**	0.001	0.001	1.864	0.076	0.182	0.136	0.038	0.248	0.242	0.399
		**Progesterone**	0.003	0.004	0.594	0.558	0.773	0.016	0.041	0.323	0.123	0.399
		**Ratio**	0.002	0.004	0.647	0.524	0.755	0.019	0.041	0.116	0.589	0.663
	**VSTP3**	**Estradiol**	-0.002	0.001	-1.122	0.274	0.504	0.054	0.066	-0.223	0.292	0.399
		**Progesterone**	-0.007	0.007	-0.995	0.331	0.568	0.043	0.067	-0.242	0.254	0.399
		**Ratio**	-0.006	0.006	-0.945	0.355	0.581	0.039	0.067	-0.216	0.31	0.399
**Oral Contraceptives**	**VSTP1**	**Estradiol**	0.006	0.002	3.146	***0.005***	***0.023***	0.301	0.104	0.424	***0.035***	0.078
		**Progesterone**	-0.045	0.090	-0.497	0.624	0.790	0.11	0.124	-0.271	0.190	0.285
		**Ratio**	-0.037	0.014	-2.684	***0.013***	***0.046***	0.239	0.108	-0.448	***0.026***	0.077
	**VSTP2**	**Estradiol**	0.003	0.001	2.486	***0.021***	0.063	0.212	0.065	0.346	0.090	0.163
		**Progesterone**	-0.103	0.049	-2.123	***0.045***	0.125	0.164	0.067	-0.48	***0.015***	0.074
		**Ratio**	-0.021	0.008	-2.669	***0.014***	***0.046***	0.236	0.064	-0.478	***0.017***	0.074
	**VSTP3**	**Estradiol**	0.009	0.002	0.004	0.997	0.997	0.006	0.006	-0.056	0.790	0.884
		**Progesterone**	0.047	0.104	0.448	0.658	0.790	0.009	0.143	0.031	0.884	0.884
		**Ratio**	0.006	0.018	0.344	0.734	0.823	0.005	0.143	0.042	0.844	0.884
**Typical Cycle**	**VSTP1**	**Estradiol**	0.002	0.003	0.562	0.580	0.773	0.014	0.097	0.075	0.720	0.756
		**Progesterone**	0.021	0.006	3.504	***0.002***	***0.010***	0.348	0.079	0.642	***0.001***	***0.005***
		**Ratio**	0.015	0.004	3.668	***0.001***	***0.007***	0.369	0.078	0.587	***0.002***	***0.011***
	**VSTP2**	**Estradiol**	0.000	0.001	-0.087	0.931	0.958	0.000	0.053	0.083	0.692	0.756
		**Progesterone**	0.006	0.004	1.431	0.166	0.352	0.082	0.051	0.072	0.734	0.756

Supplementary Table 3. Results of linear regressions and Spearman correlations in volumetric spatiotemporal patterns.

			**Linear regressions**							**Spearman correlations**		
**Cycle**	**Outcome**	**Predictor**	**Estimate *β***	***SE***	***T***	***p***	p_{FDR}	R^2	***RMSE***	***rho***	***p***	p_{FDR}
		**Ratio**	0.004	0.003	1.562	0.132	0.297	0.096	0.050	0.150	0.472	0.756
	**VSTP3**	**Estradiol**	-0.007	0.003	-1.967	0.061	0.157	0.144	0.120	-0.301	0.143	0.430
		**Progesterone**	-0.003	0.010	-0.276	0.785	0.856	0.003	0.129	-0.194	0.353	0.756
		**Ratio**	-0.003	0.007	-0.478	0.637	0.790	0.010	0.129	-0.065	0.756	0.756
**28andMe (Typical) Cycle**	**VSTP1**	**Estradiol**	0.000	0.003	-0.188	0.852	0.902	0.001	0.076	0.066	0.729	0.834
		**Progesterone**	0.017	0.004	3.891	***<0.001***	***<0.001***	0.351	0.062	0.586	***0.001***	***0.002***
		**Ratio**	0.011	0.002	4.898	***<0.001***	***<0.001***	0.461	0.056	0.693	***<0.001***	***<0.001***
	**VSTP2**	**Estradiol**	-0.005	0.006	-0.868	0.393	0.615	0.026	0.164	-0.055	0.774	0.834
		**Progesterone**	-0.044	0.008	-5.416	***<0.001***	***<0.001***	0.512	0.116	-0.631	***<0.001***	***<0.001***
		**Ratio**	-0.025	0.005	-5.091	***<0.001***	***<0.001***	0.481	0.120	-0.592	***0.001***	***0.002***
	**VSTP3**	**Estradiol**	0.003	0.001	2.742	***0.011***	***0.044***	0.212	0.033	0.571	***0.001***	***0.002***
		**Progesterone**	0.003	0.003	1.101	0.280	0.504	0.041	0.036	0.276	0.139	0.209
		**Ratio**	0.001	0.001	0.388	0.701	0.814	0.005	0.037	0.040	0.834	0.834

Note. To assess whether the volumetric spatiotemporal patterns were driven by gonadal hormone fluctuations, linear regression analyses were employed. VSTP1 = Volumetric spatiotemporal pattern 1. VSTP2 = Volumetric spatiotemporal pattern 2. VSTP3 = Volumetric spatiotemporal pattern 3. p_{FDR} = *p*-values after False Discovery Rate (FDR) correction for multiple comparisons. Significant results are indicated in bold. Estradiol = estradiol levels. Progesterone = progesterone levels. Ratio = progesterone-to-estradiol ratio.

Supplementary Table 4. Largest significant clusters in cortical thickness spatiotemporal patterns across the endometriosis cycle, the oral contraceptives cycle, the typical cycle, and the 28andMe (typical) cycle.

Region	CSTP1	CSTP2
**Cuneus**	---	409
**Entorhinal**	176	207
**Fusiform**	---	456
**Inferior parietal**	---	228
**Inferior temporal**	---	166
**Insula**	1152	1190
**Isthmuscingulate**	121	302
**Lateral occipital**	---	924
**Lateral orbitofrontal**	338	409
**Lingual**	---	1037
**Medial orbitofrontal**	---	226
**Middlet emporal**	119	---
**Paracentral**	121	---
**Parahippocampal**	288	639
**Pericalcarine**	---	642
**Posterior cingulate**	---	158
**Precentral**	435	212
**Precuneus**	---	325
**Superior temporal**	416	233

Note. Singular Value Decomposition (SVD) analysis was employed to extract cortical thickness spatiotemporal patterns (CSTP). SVD decomposed the images into spatiotemporal components, reflecting patterns of brain structure over time. To capture shared spatial patterns across individuals, data from all cycles were concatenated. Only clusters with ≥ 50 vertices are reported. Largest clusters are indicated in purple. CSTP1 = cortical thickness spatiotemporal pattern 1. CSTP2 = cortical thickness spatiotemporal pattern 2.

Supplementary Table 5. Results of Generalized Additive Models (GAMs) in cortical thickness spatiotemporal patterns.

Cycle	Term	Outcome	edf	*F*	*p*	p_{FDR}	R^2	*Deviance explained*
**Endometriosis**	**s(Day)**	**CSTP1**	2.278	3.1	0.054	0.106	0.251	32.5%
		**CSTP2**	1.0	0.0	0.934	0.934	-0.045	0.0%
**Oral Contraceptives**	**s(Day)**	**CSTP1**	1.0	3.3	0.081	0.130	0.099	12.7%
		**CSTP2**	1.0	5.7	0.026	0.101	0.162	19.7%
**Typical Cycle**	**s(Day)**	**CSTP1**	1.0	0.0	0.880	0.934	-0.042	0.1%
		**CSTP2**	1.847	1.3	0.288	384	0.081	15.2%
**28andMe (Typical) Cycle**	**s(Day)**	**CSTP1**	1.0	4.8	0.038	0.101	0.114	14.5%
		**CSTP2**	3.183	9.4	***<0.001***	***<0.001***	0.56	60.9%

Note. GAMs were performed to assess whether cortical thickness spatiotemporal patterns (CSTP1, CSTP2) fluctuated across the monthly period. The term s(Day) represents a smooth function of Day, modeling potential nonlinear fluctuations over time. The effective degrees of freedom (edf) indicate the complexity of the smooth term, with higher values suggesting greater flexibility. p_{FDR} = *p*-values after False Discovery Rate (FDR) correction for multiple comparisons. Significant results are indicated in bold.

Supplementary Table 6. Results of linear regressions and Spearman correlations in cortical thickness spatiotemporal patterns.

			Linear regressions							Spearman correlations		
**Cycle**	**Outcome**	**Predictor**	**Estimate β**	***SE***	***T***	***p***	p_{FDR}	R^2	***RMSE***	***rho***	***p***	p_{FDR}
**Endometriosis**	**CSTP1**	**Estradiol**	0.001	0.001	0.601	0.554	0.700	0.346	0.088	0.048	0.825	0.834
		**Progesterone**	0.008	0.006	1.418	0.170	0.319	0.070	0.105	0.482	***0.018***	0.109
		**Ratio**	0.008	0.005	1.783	0.088	0.214	0.029	0.107	0.393	0.058	0.175
	**CSTP2**	**Estradiol**	-0.008	0.001	-0.149	0.883	0.883	0.136	0.038	-0.045	0.834	0.834
		**Progesterone**	-0.003	0.003	-1.213	0.238	0.408	0.016	0.041	-0.096	0.656	0.834
		**Ratio**	-0.003	0.002	-1.409	0.173	0.319	0.019	0.041	-0.141	0.510	0.834
**Oral Contraceptives**	**CSTP1**	**Estradiol**	0.002	0.001	2.265	***0.033***	0.113	0.301	0.104	0.462	***0.020***	***0.030***
		**Progesterone**	-0.045	0.047	-0.971	0.342	0.547	0.11	0.124	-0.280	0.175	0.209
		**Ratio**	-0.018	0.007	-2.366	***0.027***	0.108	0.239	0.108	-0.467	***0.020***	***0.030***
	**CSTP2**	**Estradiol**	0.003	0.001	2.868	***0.009***	0.053	0.212	0.065	0.494	***0.012***	***0.030***
		**Progesterone**	-0.015	0.048	-0.305	0.763	0.832	0.164	0.067	-0.102	0.628	0.628
		**Ratio**	-0.021	0.007	-2.980	***0.007***	0.053	0.236	0.064	-0.528	***0.007***	***0.030***
**Typical Cycle**	**CSTP1**	**Estradiol**	-0.002	0.001	-2.766	***0.011***	0.053	0.014	0.097	-0.484	***0.014***	0.085
		**Progesterone**	0.004	0.002	1.492	0.149	0.319	0.348	0.079	0.185	0.375	0.750
		**Ratio**	0.003	0.002	1.851	0.077	0.214	0.369	0.078	0.339	0.098	0.293
	**CSTP2**	**Estradiol**	-0.001	0.002	-0.812	0.425	0.600	0.000	0.053	-0.053	0.801	0.835
		**Progesterone**	-0.002	0.005	-0.384	0.705	0.832	0.082	0.051	-0.088	0.675	0.835
		**Ratio**	-0.001	0.003	-0.210	0.836	0.872	0.096	0.050	-0.044	0.835	0.835
**28andMe (Typical) Cycle**	**CSTP1**	**Estradiol**	-0.010	0.006	-1.762	0.089	0.214	0.001	0.076	-0.300	0.107	0.161
		**Progesterone**	-0.011	0.012	-0.914	0.368	0.552	0.351	0.062	-0.157	0.408	0.490
		**Ratio**	-0.002	0.007	-0.307	0.761	0.832	0.461	0.056	-0.048	0.801	0.801
	**CSTP2**	**Estradiol**	0.004	0.006	0.739	0.466	0.621	0.026	0.164	0.329	0.076	0.152
		**Progesterone**	0.042	0.009	4.867	***<0.001***	***<0.001***	0.512	0.116	0.593	***0.001***	***0.002***
		**Ratio**	0.023	0.005	4.516	***<0.001***	***<0.001***	0.481	0.120	0.612	***0.001***	***0.002***

Note. To assess whether the cortical thickness spatiotemporal patterns were driven by gonadal hormone fluctuations, linear regression analyses were employed. CSTP1 = Cortical thickness spatiotemporal pattern 1. CSTP2 = Cortical thickness spatiotemporal pattern 2. p_{FDR} = *p*-values after False Discovery Rate (FDR) correction for multiple comparisons. Significant results are indicated in bold. Estradiol = estradiol levels. Progesterone = progesterone levels. Ratio = progesterone-to-estradiol ratio.

Supplementary Table 7. Significant clusters for positive associations with hormone levels in voxel-wise analyses.

	Cluster Size (voxel)								
	Estradiol			Progesterone			Ratio		
**Region**	**Endometriosis**	**Oral Contraceptives**	**All**	**Typical Cycle**	**28andMe (typical) Cycle**	**All**	**Typical Cycle**	**28andMe (typical) Cycle**	**All**
**Angular gyrus**	273	627	2535	222	1882	2451	573	2744	4072
**Anterior cingulate gyrus**	143	203	---	---	---	---	---	---	---
**Anterior insula**	---	332	---	---	---	---	---	---	---
**Calcarine cortex**	---	394	---	291	1540	817	213	1646	---
**Cerebellum**	---	615	11338	585	6158	17995	770	6585	16281
**Cuneus**	---	470	---	1230	1541	1639	1138	1098	2035
**Entorhinal area**	---	---	193	---	136	---	---	---	---
**Frontal pole**	717	---	---	---	---	---	---	---	---
**Fusiform gyrus**	---	664	1758	138	997	1634	---	1099	2034
**Gyrus rectus**	---	---	---	---	---	---	---	---	---
**Hippocampus**	---	---	181	---	431	---	---	---	---
**Inferior frontal gyrus**	679	1079	---	---	409	---	---	282	---
**Inferior occipital gyrus**	---	---	---	---	1159	820	191	1646	1019
**Inferior temporal gyrus**	---	579	1701	228	1435	2451	285	1295	4070
**Lingual gyrus**	---	556	---	117	1546	817	---	2194	2035
**Medial frontal cortex**	108	450	---	---	---	---	---	---	---
**Middle cingulate gyrus**	124	221	---	---	---	---	---	---	---
**Middle frontal gyrus**	2108	1047	5062	---	2380	4119	---	3520	5138
**Middle occipital gyrus**	---	---	---	393	179	---	569	---	---
**Middle temporal gyrus**	---	2620	5064	---	1100	1641	213	1723	5090
**Operculum**	---	1397	844	304	---	---	345	---	---
**Orbital gyrus**	1160	373	---	---	387	---	---	---	---
**Parahippocampal gyrus**	---	220	126	---	106	---	---	---	---
**Parietal operculum**	---	511	---	---	---	---	---	---	---

**Planum polare**	---	181	---	---	---	---	---	---	---
**Planum temporale**	---	160	---	---	---	---	---	---	---
**Postcentral gyrus**	754	---	844	154	403	867	164	575	225
**Posterior cingulate gyrus**	163	590	---	108	---	---	142	---	---
**Precentral gyrus**	1599	166	3373	160	---	---	195	265	1393
**Precuneus**	993	1473	4216	1691	4233	4901	1936	4938	6107
**Putamen**	---	---	---	---	---	---	---	---	---
**Superior frontal gyrus**	1995	460	5065	---	1026	2495	---	2199	1251
**Superior occipital gyrus**	110	---	---	752	102	---	783	---	---
**Superior parietal lobule**	1344	---	2536	361	2310	3267	401	3294	4074
**Superior temporal gyrus**	261	2380	3372	---	---	117	---	---	3052
**Supplementary motor cortex**	437	---	---	---	1925	---	---	2194	---
**Supramarginal gyrus**	120	390	1690	---	1138	824	---	1650	2036
**Temporal pole**	---	657	916	---	606	---	---	588	1018
**Thalamus**	---	---	214	383	---	1634	438	---	---
**Transverse temporal gyrus**	---	351	---	---	---	---	---	---	---

Note. To directly link hormonal fluctuations to volumetric brain measures, complementary voxel-wise analyses were conducted as a sensitivity check. To confirm the hormone-volumetric spatiotemporal pattern associations, we repeated the analyses at the voxel level (for volume) to assess whether similar spatial patterns of associations emerged. Voxel-wise analyses revealed widespread positive associations between brain volume and hormonal concentrations of estradiol, progesterone, and the progesterone-to-estradiol ratio (Ratio) across all individuals. These associations overlapped with the spatial patterns observed in the singular value decomposition (SVD) analyses. Contrasted analyses indicated that the endometriosis and oral contraceptive cycles predominantly drove the associations with estradiol levels, while associations with progesterone levels were primarily influenced by the typical and 28andMe (typical) cycles. Only clusters with ≥ 100 voxels are reported. *p*-Values were thresholded, $p = 0.01 – 0.0001$. Significant voxels across all individuals in all hormones are indicated in blue. Significant voxels with estradiol in the endometriosis and oral contraceptives cycle are indicated in purple. Significant voxels with progesterone in the typical and the 28andMe (typical) cycle are indicated in pink. Significant voxels with progesterone-to-estradiol ratio in the typical and the 28andMe (typical) cycle are indicated in red.

Supplementary Table 8. Significant clusters for negative associations with hormone levels in voxel-wise analyses.

	Cluster Size (voxel)			
	Progesterone	Ratio		
**Region**	**28andMe (typical) Cycle**	**28andMe (typical) Cycle**	**Endometriosis**	**Oral Contraceptives**
**Angular gyrus**	---	---	---	331
**Anterior insula**	---	---	---	326
**Calcarine cortex**	---	---	---	548
**Cerebellum**	867	768	243	935
**Cuneus**	---	---	---	877
**Fusiform gyrus**	---	---	---	735
**Gyrus rectus**	502	374	---	---
**Inferior frontal gyrus**	---	---	---	649
**Inferior temporal gyrus**	---	---	---	668
**Lingual gyrus**	---	---	---	378
**Middle frontal gyrus**	---	---	---	245
**Middle temporal gyrus**	---	---	---	2423
**Operculum**	---	---	---	1125
**Parahippocampal gyrus**	---	---	---	163
**Parietal operculum**	---	---	---	548
**Planum polare**	---	---	---	163
**Planum temporale**	---	---	---	273
**Posterior cingulate gyrus**	---	---	---	248
**Precuneus**	---	---	---	961
**Putamen**	---	---	---	490
**Superior temporal gyrus**	---	---	---	2044
**Supramarginal gyrus**	---	---	---	313
**Temporal pole**	---	---	---	318
**Transverse temporal gyrus**	---	---	---	347

Note. To directly link hormonal fluctuations to volumetric brain measures, complementary voxel-wise analyses were conducted as a sensitivity check. To confirm the hormone-volumetric spatiotemporal pattern associations, we repeated the analyses at the voxel level (for volume) to assess whether similar spatial patterns of associations emerged. Voxel-wise analyses revealed only a few negative associations, primarily observed in the oral contraceptives cycle for the progesterone-to-estradiol ratio (Ratio). Only clusters with ≥ 100 voxels are reported. *p*-Values were thresholded, p = 0.01 – 0.0001.

Supplementary Table 9. Significant clusters for associations with hormone levels in vertex-wise analyses.

Region	Cluster Size (vertex)								
	Estradiol levels				Progesterone levels			Ratio	
	Endometriosis	Oral Contraceptives	All	Typical Cycle	28andMe (typical) Cycle	All	Typical Cycle	28andMe (typical) Cycle	All
**Postcentral**	1167	---	---	---	---	---	---	---	---
**Superior parietal**	554	---	---	---	---	---	---	---	---
**Precentral**	839	---	---	---	---	---	---	---	---
**Superior frontal**	157	---	---	---	---	---	---	---	---
**Paracentral**	---	---	---	---	---	---	---	---	---
**Parahippocampal**	---	---	---	---	68	---	---	108	74
**Isthmuscingulate**	---	---	---	---	---	---	---	---	---
**Linugal**	---	---	---	---	100	---	---	125	---
**Lateral occipital**	---	---	---	---	422	---	---	360	79
**Pericalcarine**	---	---	---	---	214	---	---	206	---
**Cuneus**	---	---	---	---	159	---	---	139	---

Note. To directly link hormonal fluctuations to cortical thickness brain measures, complementary vertex-wise analyses were conducted as a sensitivity check. To confirm the hormone-cortical thickness spatiotemporal pattern associations, we repeated the analyses at the vertex level (for cortical thickness) to assess whether similar spatial patterns of associations emerged. Vertex-wise analyses revealed only a few associations between cortical thickness and hormone concentrations. Only clusters with $\geq$ 50 vertices are reported. *p*-Values were thresholded, p = 0.01 – 0.0001. Ratio = progesterone-to-estradiol ratio.

Supplementary Table 10. Results of Generalized Additive Models (GAMs) in volumetric and cortical thickness spatiotemporal patterns in the male participant.

Cycle	Term	Outcome	edf	*F*	*p*	p_{FDR}	R^2	*Deviance explained*
**Male**	**s(Day)**	**VSTP1**	8.825	848.1	***<0.001***	***<0.001***	0.997	99.8%
		**VSTP2**	8.531	67.5	***<0.001***	***<0.001***	0.962	97.5%
		**VSTP3**	8.488	427.6	***<0.001***	***<0.001***	0.994	99.6%
		**CSTP1**	1.0	0.539	0.470	0.470	-0.020	2.3%
		**CSTP2**	1.0	2.496	0.128	0.192	0.059	9.8%
		**CSTP3**	1.759	1.681	0.208	0.250	0.101	16.7%

Note. GAMs were performed to assess whether volumetric spatiotemporal patterns (VSTP1, VSTP2, VSTP3) and the cortical thickness spatiotemporal patterns (CSTP1, CSTP2, CSTP3) fluctuated across the monthly period in the male participant. The term s(Day) represents a smooth function of Day, modeling potential nonlinear fluctuations over time. The effective degrees of freedom (edf) indicate the complexity of the smooth term, with higher values suggesting greater flexibility. p_{FDR} = *p*-values after False Discovery Rate (FDR) correction for multiple comparisons. Significant results are indicated in bold.

Supplementary Table 11. Results of linear regressions and Spearman correlations in volumetric and cortical thickness spatiotemporal patterns in the male participant.

			**Linear regressions**							**Spearman correlations**		
**Cycle**	**Outcome**	**Predictor**	**Estimate**	***SE***	***T***	***p***	p_{FDR}	R^2	***RMSE***	***rho***	***p***	p_{FDR}
**Male**	**VSTP1**	**Estradiol**	0.026	0.055	0.478	0.637	0.956	0.010	0.199	0.102	0.626	0.704
		**Progesterone**	0.017	0.177	0.097	0.924	0.967	0.000	0.200	-0.202	0.332	0.541
		**Ratio**	0.002	0.058	0.042	0.967	0.967	0.000	0.200	-0.235	0.256	0.541
	**VSTP2**	**Estradiol**	0.004	0.055	0.078	0.938	0.967	0.000	0.200	-0.032	0.881	0.881
		**Progesterone**	-0.146	0.174	-0.839	0.410	0.792	0.030	0.197	0.158	0.450	0.579
		**Ratio**	-0.045	0.057	-0.785	0.440	0.792	0.026	0.197	0.190	0.361	0.541
	**VSTP3**	**Estradiol**	-0.063	0.054	-1.169	0.254	0.792	0.056	0.194	-0.366	0.072	0.541
		**Progesterone**	-0.222	0.171	-1.301	0.206	0.792	0.069	0.193	-0.232	0.264	0.541
		**Ratio**	-0.054	0.057	-0.956	0.349	0.792	0.038	0.196	-0.205	0.323	0.541
	**CSTP1**	**Estradiol**	-0.036	0.055	-0.663	0.514	0.578	0.019	0.198	-0.262	0.206	0.427
		**Progesterone**	0.175	0.173	1.009	0.323	0.477	0.042	0.196	0.384	0.059	0.265
		**Ratio**	0.058	0.057	1.019	0.319	0.477	0.043	0.196	0.508	***0.010***	0.090
	**CSTP2**	**Estradiol**	-0.027	0.055	-0.482	0.635	0.635	0.010	0.199	-0.246	0.237	0.427
		**Progesterone**	-0.224	0.171	-1.312	0.203	0.477	0.070	0.193	-0.022	0.919	0.919
		**Ratio**	-0.060	0.056	-1.058	0.301	0.477	0.046	0.195	0.100	0.633	0.712
	**CSTP3**	**Estradiol**	0.050	0.055	0.913	0.371	0.477	0.035	0.196	0.172	0.412	0.530
		**Progesterone**	-0.298	0.166	-1.801	0.085	0.382	0.124	0.187	-0.185	0.375	0.530
		**Ratio**	-0.106	0.053	-1.982	0.060	0.382	0.146	0.185	-0.27	0.191	0.427

Note. To assess whether the volumetric and cortical thickness spatiotemporal patterns were driven by gonadal hormone fluctuations, linear regression analyses were employed. VSTP = volumetric spatiotemporal pattern. CSTP = Cortical thickness spatiotemporal pattern. p_{FDR} = *p*-values after False Discovery Rate (FDR) correction for multiple comparisons. Significant results are indicated in bold. Estradiol = estradiol levels. Progesterone = progesterone levels. Ratio = progesterone-to-estradiol ratio.

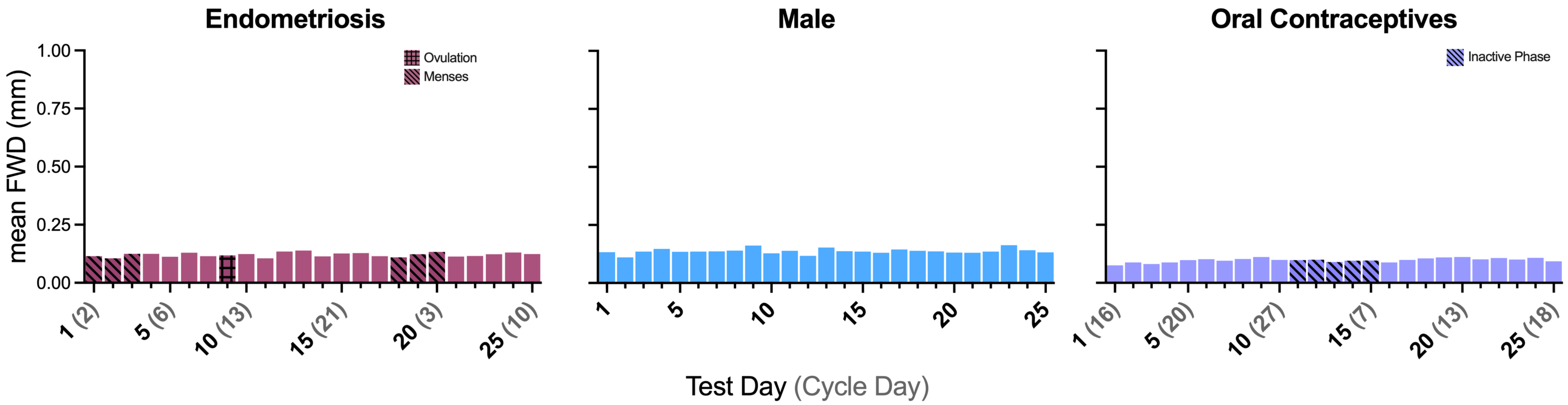

Supplementary Figure 1. Quality control for motion; mean framewise displacement (FWD) for the endometriosis cycle (*n* =1), the male (*n* =1), and the female on oral contraceptives (*n* =1). Mean FWD calculate for each test day (25) and was derived from a 12-minute resting-state functional scan acquired before T1w scans each test day and served as an indicator for motion across the entire scan duration (approximately 55 minutes). The MRI protocol included a resting-state functional scan for participants acquired in Jena, Germany, except for the typical cycle (here, the functional scan was replaced with a magnetic resonance spectroscopy scan). Mean FWD was extremely minimal across participants. Red bars indicate mean FWD for each test day in the endometriosis cycle. Blue bars indicate mean FWD for each test day in the male. Purple bars indicate mean FWD for each test day in the oral contraceptives cycle
