# Supplementary material for: Hormonal milieu influences whole-brain structural dynamics across the menstrual cycle using dense sampling in multiple individuals
Source: Nat Neurosci. 2025 Sep 26;28(12):2588–600. doi: 10.1038/s41593-025-02066-2 (PMC12672370; doi:10.1038/s41593-025-02066-2)
Supplement: Supplementary file 2 — Reporting Summary [file 41593_2025_2066_MOESM2_ESM.pdf]

nature portfolio

Corresponding author(s): Carina Heller, Friedrich Schiller University Jena, Jena, Germany

Last updated by author(s): Aug 3, 2025

Reporting Summary

Nature Portfolio wishes to improve the reproducibility of the work that we publish. This form provides structure for consistency and transparency in reporting. For further information on Nature Portfolio policies, see our Editorial Policies and the Editorial Policy Checklist.

Statistics

For all statistical analyses, confirm that the following items are present in the figure legend, table legend, main text, or Methods section.

n/a	Confirmed	
☐	☒	The exact sample size (*n*) for each experimental group/condition, given as a discrete number and unit of measurement
☐	☒	A statement on whether measurements were taken from distinct samples or whether the same sample was measured repeatedly
☐	☒	The statistical test(s) used AND whether they are one- or two-sided<br>*Only common tests should be described solely by name; describe more complex techniques in the Methods section.*
☒	☐	A description of all covariates tested
☐	☒	A description of any assumptions or corrections, such as tests of normality and adjustment for multiple comparisons
☐	☒	A full description of the statistical parameters including central tendency (e.g. means) or other basic estimates (e.g. regression coefficient) AND variation (e.g. standard deviation) or associated estimates of uncertainty (e.g. confidence intervals)
☒	☐	For null hypothesis testing, the test statistic (e.g. *F*, *t*, *r*) with confidence intervals, effect sizes, degrees of freedom and *P* value noted<br>*Give P values as exact values whenever suitable.*
☒	☐	For Bayesian analysis, information on the choice of priors and Markov chain Monte Carlo settings
☒	☐	For hierarchical and complex designs, identification of the appropriate level for tests and full reporting of outcomes
☐	☒	Estimates of effect sizes (e.g. Cohen's *d*, Pearson's *r*), indicating how they were calculated

Our web collection on statistics for biologists contains articles on many of the points above.

Software and code

Policy information about availability of computer code

Data collection

The imaging dataset for the typical cycle was acquired on a 3T MRI scanner (Prisma, Siemens Medical Solutions, Erlangen, Germany) with software version MR E11.

The imaging datasets for the endometriosis cycle, male, and female on oral contraceptives were acquired on a 3T MRI scanner (Prisma, Siemens Medical Solutions, Erlangen, Germany) with software version MR XA30.

For the 28andMe (typical) cycle dataset, scans were collected on a 3T MRI scanner (Prisma, Siemens Medical Solutions, Erlangen, Germany) with software version MR D13D.

All protocols were standard Siemens protocols. No other custom software was used for data collection.

Data analysis

- RStudio (version 2024.04.1+748)
- 'mgcv' package (version 1.9-1)
- 'stats' package (version 4.4.0)
- Statistical Package for Social Sciences (SPSS) version 27
- dcm2niix (version v1.0.20170724)
- Matlab (The MathWorks Inc., Natick, MA, USA, version R2921b)
- SPM12 (http://www.fil.ion.ucl.ac.uk/spm, version r7771)
- CAT12 (https://neuro-jena.github.io/cat, version 12.9)

For manuscripts utilizing custom algorithms or software that are central to the research but not yet described in published literature, software must be made available to editors and reviewers. We strongly encourage code deposition in a community repository (e.g. GitHub). See the Nature Portfolio guidelines for submitting code & software for further information.

Data

Policy information about availability of data

All manuscripts must include a data availability statement. This statement should provide the following information, where applicable:

- Accession codes, unique identifiers, or web links for publicly available datasets
- A description of any restrictions on data availability
- For clinical datasets or third party data, please ensure that the statement adheres to our policy

Data availability: Datasets acquired in Jena, Germany, are available at https://openneuro.org/datasets/ds006491. The dataset acquired in Santa Barbara, CA, USA, is available at https://openneuro.org/datasets/ds002674.

Research involving human participants, their data, or biological material

Policy information about studies with human participants or human data. See also policy information about sex, gender (identity/presentation), and sexual orientation and race, ethnicity and racism.

Reporting on sex and gender	In our manuscript we consistently use the terms "sex" and "female" and "male" to refer to our study population, as our central focus was on gonadal hormones as a biological varibale and how these hormones (estradiol and progesterone) impact brain structure. Sex of all participants was reported (4 females, 1 male).
Reporting on race, ethnicity, or other socially relevant groupings	In the method's section of our manuscript we report about the ethnicity of our participants, which was self reported by all participants. All subjects were white.
Population characteristics	All participants were of reproductive age (range: 23 - 37 years of age). Females recruited with a typical natural menstrual cycle (authors L.C. and L.P.) reported a regular menstrual cycle lengths (around approximately 28 days). The female diagnosed with endometriosis received the diagnosis seven months prior to the assessments (October 28, 2022) after a cyst surgery in the pelvic area. The participant was tracking her menstrual cycle length and reported a mean menstrual cycle length of 24.4 days (SD = 1.67, range = 23 – 27 days). The female on oral contraceptives (author C.H.) was using the regimen for at least three months prior to the assessment. Hormonal values for the male (author T.L.J.) were within the usual range for males. All participants reported no history of psychiatric and neurological disorders, breastfeeding or pregnancy, alcohol or drug abuse.
Recruitment	The female with endometriosis was recruited as a voluntary participant from the general population with the use of advertisements. The females with the natural menstrual cycles (authors L.C. and L.P.), the female on oral contraceptives (author C.H.), and the male (author T.L.J.) were recruited as volunteers from the Departement of Clinical Psychology, Friedrich Schiller University Jena, Jena, Germany, and the Department of Psychiatry and Psychotherapy, Jena University Hospital, Jena, Germany, as well as the Department of Psycholoical and Brain Sciences, University of California Santa Barbara, Santa Barbara, CA, USA. The project was conceived by the authors to use themselves as participants, as has been done in previous "dense-sampling" studies (cf. Poldrack et al., 2015; Pritschet et al., 2020; Heller et al., 2024). Because the authors served as participants, there is a potential for self-selection bias, as they may not represent the general population. This limits generalizability but does not affect the internal validity of the dense-sampling, within-subject design.
Ethics oversight	All participants gave written informed consent. The Friedrich Schiller University Jena Ethics Committee (for participants acquired in Jena, Germany) and the University of California, Santa Barbara Human Subjects Committee (for participant acquired in Santa Barbara, USA) approved the study. Participants were not compensated.

Note that full information on the approval of the study protocol must also be provided in the manuscript.

Field-specific reporting

Please select the one below that is the best fit for your research. If you are not sure, read the appropriate sections before making your selection.

☒ Life sciences ☐ Behavioural & social sciences ☐ Ecological, evolutionary & environmental sciences

For a reference copy of the document with all sections, see nature.com/documents/nr-reporting-summary-flat.pdf

Life sciences study design

All studies must disclose on these points even when the disclosure is negative.

Sample size	Sample size was N=5. No formal sample-size calculation was performed due to the single-subject design. Instead of collecting data from multiple individuals and then pooling the data to establish mean comparisons across groups, statistical power is achieved by following the same individual across a densely sampled time-scale (in our case four to five weeks). The number of test days was selected based on previously published dense-sampling studies investigating hormone-brain associations (cf Pritschet et al., 2020; Pritschet et al., 2024; Heller et al, 2024).<br><br>Endomtriosis cycle: n=1, 25 test days<br>Typical cycle: n=1, 25 test days

	28andMe (typical) cycle: n=1, 30 test days<br>Oral contraceptives cycle: n=1, 25 test days<br>Male: n=1, 25 test days
Data exclusions	One structural MRI scan from one day needed to be excluded due to artifacts in the scan. This particular scan was the 8th scan within the dataset of the female with endometriosis. Measurements from that day were excluded for all statistical analyses. The final number of test days for analyses for the endometriosis cycle was 24 test days.
Replication	This is the first study of its kind, and as such, no direct replications currently exist. However, to assess the reproducibility of our findings—specifically, the associations between hormones and spatiotemporal brain patterns—we conducted voxel-wise and vertex-wise sensitivity analyses. Voxel-wise analyses revealed positive associations between brain volume and hormone concentrations that spatially overlapped with the patterns identified in the SVD analyses. Consistent with the SVD results, estradiol-related associations were most prominent in the endometriosis cycle, whereas progesterone-related associations were more evident in the typical cycles. Vertex-wise analyses similarly showed few associations between cortical thickness and hormone concentrations, in line with the SVD findings. As an additional sensitivity check, we repeated the SVD, voxel-wise, and vertex-wise analyses in a male participant; none of the analyses showed meaningful associations with hormone levels.
Randomization	Since this study consists of a single-subject design where participants were scanned repeatedly over the time course of five weeks and were not grouped for analyses, randomization and covariate analyses were not necessary.
Blinding	Blinding was not possible in this study because the authors also served as participants and were fully aware of the experimental conditions, cycle phases, and test days.

Reporting for specific materials, systems and methods

We require information from authors about some types of materials, experimental systems and methods used in many studies. Here, indicate whether each material, system or method listed is relevant to your study. If you are not sure if a list item applies to your research, read the appropriate section before selecting a response.

Materials & experimental systems

n/a	Involved in the study
☐	☒ Antibodies
☒	☐ Eukaryotic cell lines
☒	☐ Palaeontology and archaeology
☒	☐ Animals and other organisms
☒	☐ Clinical data
☒	☐ Dual use research of concern
☒	☐ Plants

Methods

n/a	Involved in the study
☒	☐ ChIP-seq
☒	☐ Flow cytometry
☐	☒ MRI-based neuroimaging

Antibodies

Antibodies used	Anti-Estradiol-Ak~Biotin, 19.7 mL: Two biotinylated monoclonal anti-Estradiol antibodies (rabbit), 2.5 ng/mL and 4.5 ng/mL; Mesterolone, 130 ng/mL; MESb buffer, 50 mmol/L, pH 6.0; Preservative.<br>Anti-Progesterone-Ak~Biotin, 21.0 mL: Biotinylated monoclonal anti-Progesterone antibody (recombinant, sheep), 30 ng/mL; Phosphate buffer, 25 mmol/L, pH 7.0; Preservative.<br>Anti-FSH-Ab~biotin, 10 mL: Biotinylated monoclonal anti?FSH antibody (mouse) 0.5 mg/L, MES buffer 50 mmol/L, pH 6.0; preservative.<br>Anti-FSH-Ab~Ru(bpy), 10 mL: Monoclonal anti?FSH antibody (mouse) labeled with ruthenium complex 0.8 mg/L, MES buffer 50 mmol/L, pH 6.0; preservative.<br>Anti-LH-Ab~biotin, 10 mL: Biotinylated monoclonal anti?LH antibody (mouse) 2.0 mg/L; TRIS buffer 50 mmol/L, pH 8.0; preservative.<br>Anti-LH-Ab~Ru(bpy), 10 mL: Monoclonal anti?LH antibody (mouse) labeled with ruthenium complex 0.3 mg/L; TRIS buffer 50 mmol/L, pH 8.0; preservative.
Validation	Hormone concentrations were measured using standardized electrochemiluminescence immunoassays (ECLIA) on the Roche cobas e platform:<br>- Electrochemiluminescence immunoassay (ECLIA) Elecsys® Estradiol III Assay (https://diagnostics.roche.com/global/en/products/lab/elecsys-estradiol-iii-cps-000466.html): measuring range, 18.4 – 11,010 pmol/l (5 – 3000 pg/ml); intra-assay precision, ≤ 8.4% variation coefficient.<br><br>- Electrochemiluminescence immunoassay (ECLIA) Elecsys® Progesterone III Assay (https://diagnostics.roche.com/global/en/products/lab/elecsys-progesterone-iii-cps-000501.html): measuring range, 0.159 – 191 nmol/l (0.05 – 60 ng/ml); intra-assay precision, ≤ 20.7% variation coefficient.<br><br>- Electrochemiluminescence immunoassay (ECLIA) Elecsys® FSH Assay (https://diagnostics.roche.com/global/en/products/lab/elecsys-fsh-cps-000472.html): measuring range, 0.3 – 200 mIU/ml (0.3 – 200 IU/l); intra-assay precision, ≤ 2.1% variation coefficient.<br><br>- Electrochemiluminescence immunoassay (ECLIA) Elecsys® LH Assay (https://diagnostics.roche.com/global/en/products/lab/elecsys-lh-cps-000492.html): measuring range, 0.3 – 200 mIU/ml (0.3 – 200 IU/l); intra-assay precision, ≤ 2.2% variation coefficient.

All assays were determined on the cobas® e 402/801 analyzer (Roche Diagnostics GmbH, Mannheim, Germany) and were used according to the manufacturer's instructions. The reported intra-assay precision and coefficient of variation values are taken from the manufacturer's package inserts and reflect the analytical performance of the assays. These values are based on Roche's validation studies and do not represent quality control data generated at the Institute of Clinical Chemistry and Laboratory Diagnostics, Jena University Hospital, Jena, Germany.

Plants

Seed stocks	*Report on the source of all seed stocks or other plant material used. If applicable, state the seed stock centre and catalogue number. If plant specimens were collected from the field, describe the collection location, date and sampling procedures.*
Novel plant genotypes	*Describe the methods by which all novel plant genotypes were produced. This includes those generated by transgenic approaches, gene editing, chemical/radiation-based mutagenesis and hybridization. For transgenic lines, describe the transformation method, the number of independent lines analyzed and the generation upon which experiments were performed. For gene-edited lines, describe the editor used, the endogenous sequence targeted for editing, the targeting guide RNA sequence (if applicable) and how the editor was applied.*
Authentication	*Describe any authentication procedures for each seed stock used or novel genotype generated. Describe any experiments used to assess the effect of a mutation and, where applicable, how potential secondary effects (e.g. second site T-DNA insertions, mosiacism, off-target gene editing) were examined.*

Magnetic resonance imaging

Experimental design

Design type	No task or resting-state MRI acquired
Design specifications	No task or resting-state MRI acquired
Behavioral performance measures	No task or resting-state MRI acquired

Acquisition

Imaging type(s)	Structural MRI: T1-weighted (T1w) magnetization prepared - rapid gradient echo (MPRAGE) sequence with the generalized autocalibrating partially parallel acquisitions (GRAPPA) acceleration.
Field strength	3T
Sequence & imaging parameters	Data from Jena: echo time (TE) = 2.22 ms, repetition time (TR) = 2400 ms, inversion time (TI) = 1000 ms, flip angle = 8°, matrix size = 320 x 320 pixels, field of view (FOV) = 256 mm, band width = 220 Hz/pixel, and slice thickness = 0.80 mm. Data from Santa Barbara: TE = 2.31 ms, TR = 2500 ms, TI = 934 ms, flip angle = 7°, matrix size = 320 x 320 pixels, FOV = 255 mm, band width = 210 Hz/pixel, and slice thickness = 0.80 mm.
Area of acquisition	Whole brain scan

Diffusion MRI ☐ Used ☒ Not used

Preprocessing

Preprocessing software	The T1w images were converted from Dicom to Nifti files using dcm2niix (Chris Rorden, version v1.0.20170724, https://www.nitrc.org/projects/mricrogl/) and then preprocessed in SPM12 (http://www.fil.ion.ucl.ac.uk/spm) and the CAT12 (https://neuro-jena.github.io/cat) toolbox using the longitudinal pipeline approach in Matlab R2021b (The MathWorks Inc., Natick, MA, USA). All T1w images were corrected for bias-field inhomogeneities and initially tissue-classified into gray matter, white matter, and cerebrospinal fluid, followed by an adaptive maximum a posteriori segmentation, which also accounts for partial volume effects
Normalization	The resulting gray and white matter partitions were spatially normalized to MNI space Geodesic Shooting Registration.
Normalization template	MNI space Geodesic Shooting Registration
Noise and artifact removal	Please see above: One scan from the female with endometriosis had to be excluded due to artefacts within the corpus callosum and subcortical structures. All other T1-weighted images were corrected for bias-field inhomogeneities and tissue-classified into gray matter, white matter, and cerebrospinal fluid, which also included an approach accounting for partial volume effects by applying adaptive maximum a posteriori estimations and a hidden Markov Random Field Model.
Volume censoring	N/A

Statistical modeling & inference

Model type and settings	Singular Value Decomposition (SVD) was used to extract spatiotemporal patterns from the preprocessed images by decomposing the three-dimensional image sets into spatial patterns (maps) and their associated temporal dynamics (time

	course) for each participant separately. The spatial patterns represent the regions of the brain that share a similar temporal pattern, while the temporal dynamics describe how the local volume of these regions changes over time. SVD analysis was performed for all female individuals combined (group-level). SVD analysis for the male was performed separately. The SVD analyses yielded more than one spatiotemporal patterns exceeding a threshold of 1.
Effect(s) tested	We assessed the variations in whole-brain volumetric and cortical thickness spatiotemporal patterns across the monthly period using general additive modeling (GAM). This approach acknowledges the anticipated complexity and nonlinearity of the relationship between the menstrual cycle and brain structure, allowing for a more adaptable modeling of menstrual cycle-dependent trajectories in structural brain dynamics. We then employed linear regression models with volumetric and cortical thickness spatiotemporal patterns as dependent variables and the gonadal hormones as predictors. Since not all variables were normally distributed, relationships were further modeled using non-parametric functional Spearman rank correlation. Results were highly consistent across both approaches. Furthermore, to account for possible autocorrelation, GAMs with an autogressive term and functional linear regressionswith an autoregressive terms were calculated. Results were highly consistent across approaches with and without autoregressive terms. However, autoregressive terms led to overfitting of the model which supported our decision to use the simpler models.<br>To investigate the association between hormonal concentrations and structural brain measures at each voxel or vertex, statistical analysis using a general linear model (GLM) was performed. Hormonal concentrations were included as the dependent variable in a regression framework. To identify statistically significant effects, the Threshold-Free Cluster Enhancement (TFCE) method was used.

Specify type of analysis: ☒ Whole brain ☐ ROI-based ☐ Both

Statistic type for inference<br>(See Eklund et al. 2016)	Voxel-wise, vertex-wise
Correction	Corrected for multiple comparisons using False Discovery Rate (FDR) and Family-Wise Error (FWE).

Models & analysis

n/a	Involved in the study
☒	☐ Functional and/or effective connectivity
☒	☐ Graph analysis
☒	☐ Multivariate modeling or predictive analysis
